# Terpenoids, Cannabimimetic Ligands, beyond the *Cannabis* Plant

**DOI:** 10.3390/molecules25071567

**Published:** 2020-03-29

**Authors:** Elaine C. D. Gonçalves, Gabriela M. Baldasso, Maíra A. Bicca, Rodrigo S. Paes, Raffaele Capasso, Rafael C. Dutra

**Affiliations:** 1 Laboratory of Autoimmunity and Immunopharmacology (LAIF), Department of Health Sciences, Campus Araranguá, Universidade Federal de Santa Catarina, Araranguá 88906-072, Brazil; elainecdalazen@gmail.com (E.C.D.G.); baldasso.gabriela@gmail.com (G.M.B.); rodrigosebbenp@gmail.com (R.S.P.); 2 Graduate Program of Neuroscience, Center of Biological Sciences, Campus Florianópolis, Universidade Federal de Santa Catarina, Florianópolis 88040-900, Brazil; 3 Neurosurgery Department, Neurosurgery Pain Research institute, Johns Hopkins School of Medicine, Baltimore, MD 21287, USA; bicca.ma@jhmi.edu; 4 Department of Agricultural Sciences, University of Naples Federico II, 80,055 Portici, Italy

**Keywords:** phytocannabinoid, terpenoids, cannabinoid receptors, *Cannabis* plant, endocannabinoids, inflammation.

## Abstract

Medicinal use of *Cannabis sativa* L. has an extensive history and it was essential in the discovery of phytocannabinoids, including the *Cannabis* major psychoactive compound—Δ9-tetrahydrocannabinol (Δ9-THC)—as well as the G-protein-coupled cannabinoid receptors (CBR), named cannabinoid receptor type-1 (CB1R) and cannabinoid receptor type-2 (CB2R), both part of the now known endocannabinoid system (ECS). Cannabinoids is a vast term that defines several compounds that have been characterized in three categories: (i) endogenous, (ii) synthetic, and (iii) phytocannabinoids, and are able to modulate the CBR and ECS. Particularly, phytocannabinoids are natural terpenoids or phenolic compounds derived from *Cannabis sativa*. However, these terpenoids and phenolic compounds can also be derived from other plants (non-cannabinoids) and still induce cannabinoid-like properties. Cannabimimetic ligands, beyond the *Cannabis* plant, can act as CBR agonists or antagonists, or ECS enzyme inhibitors, besides being able of playing a role in immune-mediated inflammatory and infectious diseases, neuroinflammatory, neurological, and neurodegenerative diseases, as well as in cancer, and autoimmunity by itself. In this review, we summarize and critically highlight past, present, and future progress on the understanding of the role of cannabinoid-like molecules, mainly terpenes, as prospective therapeutics for different pathological conditions.

## 1. The Era of *Cannabis sativa*, Cannabinoids, and the Endocannabinoid System: A Long Journey Traveled

The *Cannabis sativa* era has a long and remarkable history dating from prehistoric Xinjiang, an ancient Chinese place, where users consumed *Cannabis* not only for religious/spiritual or hedonic purposes but also for its medicinal effects [1,2,3]. The first report of hemp medicinal use comes from Chinese medicine, around 2300 B.C. In India, *Cannabis* became part of the Hindu religion, being subsequently introduced to Europe between 1000 and 2000 B.C. Long after *Cannabis* reached the Americas, South America (mainly Chile) in 1545, and over 60 years later (1606), its cultivation was introduced to North America. Western medicine slowly progressed from the understanding and moderate use in the early and mid-19th century, to its wider use, based on its medicinal properties in the 20^th^ century. Nevertheless, due to prejudice and misinformation, the use of this plant has been marginalized, which has hindered research progress regarding its medicinal beneficial effects [1,2]. 

Currently, *Cannabis* is the most commonly cultivated, trafficked, and abused drug worldwide, potentially causing a substantial public health impact since it can alter sensory perception and induce elation and euphoria [4,5]. Recent use rates among the population in general show a concentration to adolescents and young adults (20 to 24 years-old), ranging from 2%–5% of the global population (an estimated 13 million cannabis-dependent individuals in 2010); yet, the highest numbers (∼10%–13%) are reported in North America [5,6,7]. A study published by Hasin and colleagues revealed a significant rise in marijuana use prevalence in 2001–2002 and 2012–2013, accompanied by a large increase of marijuana-induced disorders in this same time period [8,9]. Conversely, another study showed that *Cannabis*-induced disorders declined among young users during 2013-2014, in the USA [10,11]. According to United States Code, “marijuana/cannabis” comprises “all parts” of the plant *Cannabis sativa L*. and every compound derivative of such plant. By the year 2016, 28 states in the USA have voted to authorize or implement medicinal cannabis programs. Among these, eight states and the district of Columbia have legalized the recreational use of *Cannabis* [12]. In other countries, including the United Kingdom (UK), Denmark, Czech Republic, Austria, Sweden, Germany, and Spain, it is formally approved; thus, decriminalizing the therapeutic use of *Cannabis* and cannabis-based products [13,14]. Pioneering in Latin America, Uruguay, became the first country to legalize the sale, cultivation, and distribution of *Cannabis* [15,16]. Wilkinson and D’Souza have previously described that the medicalization and/or incorporation of *Cannabis* into a medicine is complex for a number of reasons, including that (i) it is a plant rather than a pharmaceutical product, and (ii) knowledge of its properties and effects is still limited [17]. However, in light of the recently and largely reported pharmacological discoveries and therapeutic benefits of *Cannabis,* the controlled and medicinal use of *Cannabis* for some pathological conditions have been enforced.

Era of cannabinoids started when Mechoulam and Gaoni isolated and characterized the main psychoactive component of *Cannabis sativa*, the Δ9- tetrahydrocannabinol (Δ9-THC). Subsequently, in 1988, Howlett’s group established the presence of a specific cannabinoid receptor in the rat brain by using a tritium labeled cannabinoid [18], followed by the cloning of the cannabinoid receptor type-1 (CB1R) [19]. Then, Matsuda and coworkers (1990) described a second receptor, named the cannabinoid receptor type-2 (CB2R), which was cloned by Munro and coworkers in 1993 [18,19]. These receptors can be activated by endogenous molecules produced normally by our bodies, and likewise by external synthetic and natural molecules. The number of natural compounds identified or isolated from *Cannabis sativa* has been increasing in the last decade, with 565 identified substances between cannabinoids and non-cannabinoid constituents [20]. The genus *Cannabis* comprises closely related species, mainly, *Cannabis indica*, *Cannabis ruderalis* (identified in 1924), *Cannabis sativa L*., which is widely known as “hemp” and not psychoactive, as well as *Cannabis sativa*, which induces psychoactive effects [1]. Cannabinoids are defined as a group of molecules that modulate cannabinoid receptors (CBR) and are characterized by three varieties, such as endogenous or endocannabinoids, synthetic cannabinoids, and phytocannabinoids. The latter variety comprehends natural terpenoids or phenolic compounds derived from *Cannabis sativa* or other species, and will be further explored later in this review [21]. Altogether, 120 cannabinoids have been isolated from the *Cannabis sativa* plant and classified into 11 general types, as described below (Table 1) [20]. 

Pharmacologically approaching, three compounds have been isolated and identified as the most important, namely the Δ9-tetrahydrocannabinol (Δ9-THC), cannabidiol (CBD), and cannabinol (CBN). Relevantly, preclinical and clinical research has shown that cannabinoids, especially CBD, play key a role in different pathological conditions (Table 2).

When we talk about the era of the “endocannabinoid system”, we have to keep in mind that this biological system was named over the response of its receptors to cannabinoid drugs, such as the previously mentioned and well-studied Δ9-THC and biologically active synthetic analogs, just like it has happened with the opioids in the past. In addition to its receptors, the system is highly modulated by the enzymes involved in the endogenous cannabinoids synthesis and inactivation (endocannabinoid metabolism). Furthermore, some other receptors have been reported to be activated by cannabinoid drugs and related molecules, including GPR55, GPR18, and GPR119 [40,41,42]. CB1R is a key component of the endocannabinoid system (ECS), since it interacts with endogenous and exogenous cannabinoids, including Δ9-THC, and it is considered the most abundant metabotropic receptor in the brain [43]. It has been cloned from humans and it is accountable for the *Cannabis* effects on mood, as well as negative psychotomimetic effects, including anxiety, paranoia, and dysphoria [4,44]. While CB1R plays a role as a neurotransmission regulator in different brain regions and for this reason mediates the *Cannabis* psychoactive effects, CB2R, in particular, mediates anti-inflammatory and immunomodulatory actions [45]. An accumulating body of evidence suggests that both CB1R and CB2R, and their ligands, play a significant role in physiologic and pathologic processes [46]. In this context, both receptors have been widely studied regarding their relevance in the modulation of immune-mediated inflammatory diseases, neuroinflammation, neurological and neurodegenerative diseases, cancer, and autoimmunity. 

Beyond the CBR, mammalian tissues can both synthesize and release cannabinoid receptor ligands [44,47,48]. The era of ECS started when Devane and colleagues (1992) described for the first time, the N-arachidonoylethanolamine molecule, named anandamide from porcine brain. Interestingly, anandamide interact to CBR and induces behavioral actions similar to the ones induced by Δ9-THC, when administered in rodents [4,49]. The mainly endogenous cannabinoids are the anandamide (AEA) and the 2-arachidonoyl glycerol (2-AG). It is now ordinarily accepted that the mammalian tissues contain an ECS composed by: (i) CB1R and CB2R cannabinoid receptors [19,44], (ii) endogenous cannabinoids ligands [49,50,51], and (iii) enzymes involved in the cannabinoids ligands synthesis and inactivation. Regarding these enzymes, the fatty acid amide hydrolase (FAAH) breaks amide bond and releases arachidonic acid and ethanolamine from AEA, and the monoacylglycerol lipase (MAGL) is responsible for a more efficiently 2-AG degradation [52]. Endocannabinoids are produced on demand from membrane lipids using the machinery of the enzymes responsible for their synthesis, transport, and degradation. For instance, the N-arachidonoyl phosphatidylethanolamine (NArPE) originates a phosphatidic acid by a reaction mediated by a specific phospholipase D (NAPE-PLD); most importantly, it is hydrolyzed to AEA, in a reaction catalyzed by N-acyltransferase (NAT). The latter reaction happens out of an acyl group from the arachidonoylphosphatidylcholine (diArPC) sn-1 position converted to a phosphatidylethanolamine (PE) amino group. Following, AEA is degraded by FAAH. Synthesis of 2-AG depends on the phosphatidylinositol (PI) conversion to diacylglycerol (DAG) by the phospholipase C (PLC) enzyme, and subsequent DAG transformation to 2-AG by the action of the diacylglycerol lipase (DAGL) [53]. The ECS is involved with multiple biological functions, such as immune-mediated inflammatory and autoimmune diseases [53], as well as neuroinflammatory and neurodegenerative conditions [54]. Moreover, the ECS participates in the immune control at the CNS [55], maintaining overall “fine-tuning” of immune response balance [56], and influencing the neuroendocrine reaction to inflammation and infection [57].

Importantly, the ECS (i.e., CBR, endogenous cannabinoids, and anabolic/catabolic enzymes) are present in the cardiovascular tissues (myocardium, smooth muscle, and vascular endothelial cells), as well as in the circulating blood cells [58]. CB1R are expressed in the peripheral nervous system, including vagal afferent neurons, while CB2R are expressed in cardiomyocytes, coronary artery endothelial cells, and smooth muscle cells. For this reason, the endocannabinoid signaling exerts complex cardiac and vascular effects ranging from vasodilatation to vasoconstriction, and decreased myocardial contractility [58]. Those are important biological effects, as they could play an essential role in side effects promoted by potential molecules that are able to modulate this system. For instance, in healthy individuals, CB1R activation decreased myocardial contractility and blood pressure, possibly by peripheral inhibition of noradrenaline release from postganglionic sympathetic axons that leads to regulation of cardiac output [59]. In an opposite way, CB2R may exert a cardioprotective role associated to its immunomodulatory properties during tissue inflammation and tissue injury in cardiovascular diseases. The endogenous cannabinoids (2-AG and AEA) also have vascular effects, which are mediated by perivascular transient receptor potential vanilloid 1 (TRPV1) and transient receptor potential vanilloid 4 (TRPV4) activation in smooth muscle cells, promoting dilatory response [60]. Between the common clinical adverse effects associated with the *Cannabis* plant use, the increased cardiovascular activity and heart rate, as well as decreased blood pressure have been described [60]. In addition, the uses of *Cannabis* plant or synthetic cannabinoids have been linked to myocardial infarction, cardiomyopathy, arrhythmias, and stroke [58,61,62]. It occurs, possibly due to dose-dependent effects of phytocannabinoids and consequent modulation of the autonomic nervous system, at least partly via CB1R activation [60], since the CB1R antagonist Rimonabant^®^ ameliorate the cannabis-induced tachycardia [63,64]. It is important to be aware of the harmful consequences that come along with the use of *Cannabis* plant and/or synthetic cannabinoids, as they could contribute to development of cardiovascular disorders, since the ECS has an essential role in the cardiovascular signaling.

**Figure 1:**
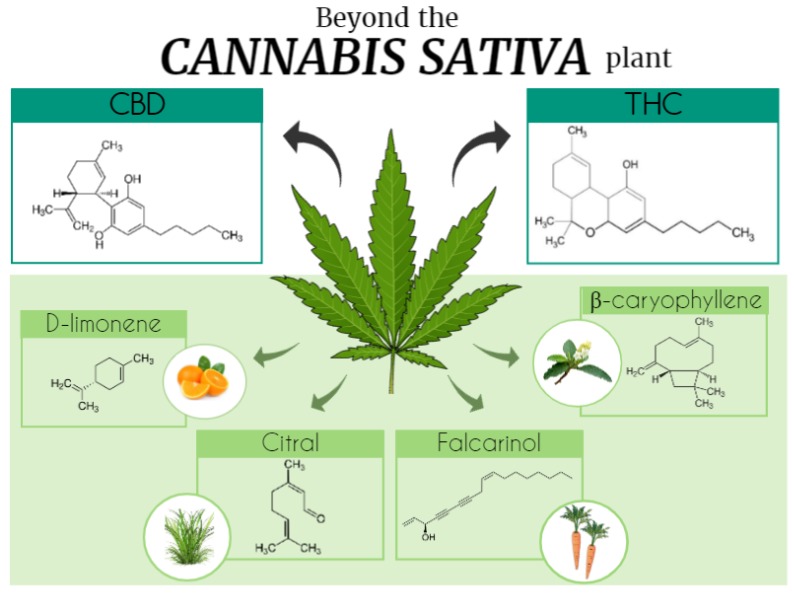
Beyond the *Cannabis sativa* plant. The Era of cannabinoids started with the description and isolation of the main *Cannabis sativa* psychoactive component, Δ9-tetrahydrocannabinol (THC). However, many other natural compounds were also identified, totalizing 565 substances among cannabinoids and non-cannabinoids constituents. This figure illustrates some of the *Cannabis sativa* compounds (d-limonene, β-caryophyllene, citral, and falcarinol) and its molecular structures that can be also found in other plants, such as *Cordia verbenacea*, lemon, *Cymbopogon citratus*, and carrot. CBD, cannabidiol. Figure created using the Mind the Graph platform.

The future, shedding light to a new era, is promising and based on the cloning of CBR associated with the possibility of manipulation of endocannabinoid levels in tissues, by using endocannabinoid enzymes-targeted pharmacology. This represents an opening of a possible gateway to the discovery and/or development of cannabimimetic ligands, beyond the *Cannabis* plant, which could still show therapeutic effects and possibly rule out many of the important adverse effects. A previous review has already stated that some plants, not belonging to the *Cannabis* genus, produce molecules chemically similar to the phytocannabinoids, named cannabimimetic ligands [65] (Figure 1). Cannabinoid-like molecules (mainly terpenes) of either plant or synthetic origin that are non-psychotropic have been studied. Terpenes and terpenoids are a widespread group of secondary metabolites found in numerous plant families, including Cannabaceae and others. Herein, we discuss the role of cannabinoid-like molecules, mainly terpenes, as prospective therapeutics for a variety of pathological conditions.

## 2. Cannabis Phytocannabinoids: Focus on Tetrahydrocannabinol and Cannabidiol

The phytocannabinoid class includes more than a 100 compounds that are present in the *Cannabis sativa* plant [66], which interact with components of the human ECS, briefly addressed in this section. Phytocannabinoids production is dependent on plant internal factors (synthesized hormone levels, plant kind, and parts of the plant) and on external factors (humidity, light, type of soil, and temperature). The most elucidated compounds among the main phytocannabinoids are CBN, CBD, ∆8- e ∆9-THC, cannabigerol, and cannabivarin. The ∆9-THC is the major psychotropic compound found in high concentrations in the *Cannabis sativa* plants. It is classified as a CB1R and CB2R partial agonist, showing preference for the CB1R. The agonist activity on CBR triggers adenylyl cyclase (AC) inhibition and, thereby, the ability of modulating different neurotransmitters release as dopamine, acetylcholine, glutamate, and gamma-aminobutyric acid (GABA) [66]. Of note, phytocannabinoids not only bind to CBR, but also show potential actions on different kinds of receptors, such as peroxisome proliferator-activated receptors (PPAR), glycine receptors, and the transient receptor potential (TRP) cation channels. The CBD, unlike the tetrahydrocannabinol (THC), is a non-psychotropic cannabinoid that has been widely investigated regarding its potential therapeutic use. It has been already established in the literature that CBD shows anti-inflammatory, anti-epileptic, analgesic, anxiolytic, and neuroprotective properties, as well as it can be used to mitigate Parkinson’s disease (PD) symptoms [67,68,69]—Table 2. CBD acts as a negative allosteric modulator of CB1R [65] and as an inverse agonist in CB2R, besides being a FAAH enzyme inhibitor.

To briefly highlight, many other phytocannabinoids (e.g., cannabigerol, cannabichromene, and cannabinol) showed significant therapeutic value. The cannabigerol (CBG) showed agonist and antagonist activity on TRP channels and it was also able to produce 5-HT_1_ and CB1R antagonism [70]. Additionally, CBG is an AEA reuptake inhibitor [71], and it showed colon anti-tumor activity by inhibiting transient receptor potential melastatin 8 (TRPM8) channels [72]. Relevantly, when associated with CBD, it demonstrated anti-inflammatory activity reducing tumor necrosis factor (TNF) expression and upregulating Interleukin–10 (IL-10) and Interleukin–37 (IL-37) levels [70]. Cannabichromene (CBC) showed agonist activity on CB2R [73]. Besides, it interacts with TRP channels, being suggested as a potential therapeutic resource for the treatment of pain and inflammation [71]. Lastly, CBN showed similar therapeutic properties to other phytocannabinoids, such as anticonvulsant, anti-inflammatory, and antibacterial [71]. In addition, CBN showed inhibitory activity on cyclooxygenase (COX), lipoxygenase (LOX), and P450 cytochrome enzymes [71], as well as on keratinocyte proliferation, supporting a possible potential therapeutic for psoriasis cases [74]. As it can be appreciated with the major phytocannabinoids, the wide ranges of possible interactions of these molecules with multiple targets in our body, demonstrates the magnitude and the complexity of phytocannabinoids acting in living organisms.

**Figure 2:**
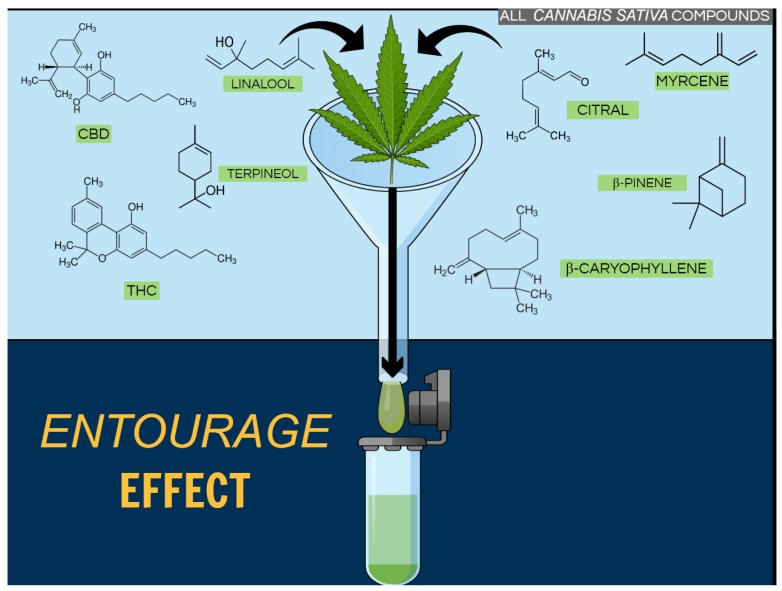
Entourage effect. Beyond the Δ9- tetrahydrocannabinol (Δ9-THC) and cannabidiol (CBD), there are many compounds present in *Cannabis sativa*, including terpenoids (such as linalool, terpineol, and citral), which could contribute to beneficial effects related to this plant. However, the underlying mechanism of these medicinal effects is largely unknown when molecules are associated. Figure created using the Mind the Graph platform.

We just established that phytocannabinoids demonstrate different pharmacological effects, and it can get even more intriguing and complex when we focus on previous data describing that the combined use of some phytocannabinoids can possibly increase the positive effects proportionate by them. For instance, the use of CBD associated with ∆9-THC promoted downregulation of the neuroinflammatory process in animal models of multiple sclerosis (MS) [75], besides, reducing pain [76] and muscle spasticity in MS patients [75]. Importantly, CBD attenuated the psychotropic effects of THC when used in a combined form [75]. This last piece of data supports the hypothesis that CBD binds to an allosteric site on CB1R that is functionally distinct from the active site for 2-AG and THC [77]. In this same context, a recent study reported that a botanical drug preparation (BDP) was more potent than pure THC to produce antitumor responses in cell culture and animal models of breast cancer. While pure THC mainly activated CB2R and generated reactive oxygen species (ROS), the BDP modulated different targets and mechanisms of action [78]. This combined effect, observed with the association of phytocannabinoids and other compounds present in the *Cannabis sativa* plant, such as terpenoids, is known as the entourage effect [79] (Figure 2).

### Cannabis Terpenoids

Beyond the phytocannabinoids, the *Cannabis* plant is able to produce a diversity of compounds. Thirty-one-years ago, Mechoulam and Ben-Shabat described what they named the ‘’entourage effect’’, suggesting interactions between *Cannabis* “inactive” metabolites and closely related molecules could markedly increase the activity of the “primary” cannabinoids (Figure 2). From this, it was possible to hypothesize that could be a contribution of “minor cannabinoids” and *Cannabis* terpenoids to the plant overall pharmacological effect. Therefore, a recent study evaluated the effect of common terpenoids, by themselves and in combination with THC, in AtT20 cells expressing CB1R or CB2R. Surprisingly, none of the analyzed terpenoids modulated the THC phytocannabinoid agonist signaling. Thus, the authors suggested that if the phytocannabinoids–terpenoids entourage effect exists, it is not at the CB1R or CB2R receptor level [80]. Corroborating, when rats were submitted to an abdominal writhing model and treated only with terpenoids they demonstrated increased abdominal writhing, while the animals treated with THC showed robust analgesia, even better than the rats that received the *Cannabis* full extract. In this case, *Cannabis* antinociceptive property was linked to Δ9-THC, since terpenes alone do not alter the nociceptive behavior [81]. Using a different approach, Nandal and co-authors exposed cancerous cell lines to treatment with phytocannabinoids combined with low concentrations of co-related terpenoids. They observed increased cell mortality at ratios similar to the ones obtained with the natural plant extracts [82]. According to the authors, their results differed from Santiago et al. findings because they evaluated terpenoids without statistical correlation to THC, meaning that terpenoids concentrations in their preparations where higher than the natural-occurred in the plants [80,82]. Thus, the possible “entourage effect” and the positive contribution derived from the addition of terpenoids to cannabinoids could be interpreted as uncertain. However, the study of terpenoids represents an open window that goes beyond its actions *(i)* in the endocannabinoid system solely, or *(ii)* as mere phytocannabinoids passive co-authors, and even beyond the *Cannabis* plant. 

## 3. Terpenoids in and beyond the *Cannabis* Plant

*Cannabis* contains a large number of monoterpene and sesquiterpene compounds, together called terpenoids or terpenes, which are aromatic compounds synthesized in trichomes [71]. In the plant, these compounds (i.e., more than 120 terpenes) synthesized alongside phytocannabinoids are important volatile constituents that are responsible for the plant’s characteristic smell and also serve for different organic functions, such as insect repellent, repellent to herbivore attack, and attractive to pollinators [71]. Booth and Bohlmann described the terpenes- and cannabinoid-rich resin as the most valuable cannabis products, with different psychoactive and medicinal properties [83]. Studies regarding terpenoid compounds (i.e., D-limonene, β-myrcene, α-pinene, α-terpineol, β-pinene, β-caryophyllene, and others) have been growing in the last decades due to their large number and extensive employability [71,84]. However, the presence of terpenoids has not been restricted to the *Cannabis sativa* plant. These compounds normally occur in several other plant species, such as *Mirabilis jalapa*, *Lithophragm glabrum*, *Cordia verbenacea*, *Eucalyptus globus*, *Syzygium aromaticum*, *Senna didymobotrya*, *Cymbopogon citratus,* and in some *Citrus* genus plants, as *Citrus limon* and others. To date, there are more than 10,000 articles versing about phytocannabinoids or cannabimimetics, and its actions described in the literature. There are many *Cannabis* terpenoid compounds that are not majorly found in the *Cannabis* plant but are highly expressed in other plants. Its actions are varied and complex, being many compounds studied deep down to the mechanisms of action, pharmacokinetics, toxicity, and pharmacodynamics, whereas others are still to be addressed regarding these aspects. The study about terpenoids beyond the *Cannabis* plant has been earning ground in the research field due to the fact that they can be utilized as tools for the improvement of therapeutic research for several diseases. Herein, we can have a sense of how literature stands at this end regarding some of these compounds, and we discuss the role of terpenoids as prospective therapeutics of different pathological conditions.

### 3.1. Beta (β)- and α-Caryophyllene

Beta and alpha-Caryophyllene are the major sesquiterpenes encountered in the *Cannabis* plant [85]. Importantly, a comparative study showed that regardless the type of extraction used supercritical fluid extraction, steam distillation, or hydrodistillation, the major sesquiterpene compound to be extracted was β-Caryophyllene (BCP) [86]. Caryophyllenes are considered phytocannabinoids with strong affinity to CB2R but not CB1R [87], and are produced not only by *Cannabis* but also by a number of plants, as a mechanism of defense to insects, for instance. The vast literature describes a number of plants that contain this compounds such as *Cordia verbenacea*, *Pterodon emarginatus*, *Artemisia campestris*, *Lantana camara*, *Centella asiatica*, *Cyanthillium cinereum*, and *Croton bonplandianus*, just to name a few of the more than 30 species previously described. Heretofore published original articles described seven main actions to caryophyllenes. These actions are reported to be repellent, antimicrobial or antibacterial, anticancer or antiproliferative, antifungal, AChE inhibitor, antioxidant, and anti-inflammatory. Regarding the antifungal and antimicrobial action, Sabulal and co-workers showed that *Zingiber nimmonii* rhizome oil, which is a unique isomeric caryophyllene-rich natural source, has inhibitory activity against fungi (e.g., *Candida glabrata*, *Candida albicans*, and *Aspergillus niger*) as well as against both *Bacillus subtilis* and *Pseudomonas aeruginosa* bacteria [88]. More recently, a study has shown that *Phoebe formosana* leaf extract has antifungal activity as well; BCP being one of the active compounds identified [89]. In this same study, authors have reported that the oil exhibited cytotoxic activity against human lung, liver, and oral cancer cells while the major active compound was BCP. Corroborating, BCP was the major compound found in the tree bark essential oil from *Pinus eldarica*, which showed antiproliferative activity in a concentration dependent manner against MCF-7 breast cancer cell line [90]. Likewise, anticancer activity against MCF-7 cells was also reported for the essential oil of *Cyperus longus* mainly constituted of β- and α- caryophyllenes [91]. Regarding analgesic effects, BCP has been demonstrated to attenuate paclitaxel (PTX)-induced peripheral neuropathy in mice by a mechanism dependent on mitogen-activated protein kinase (MAPK) inhibition [92]. Recently, a review has summarized, very well, the anticancer and analgesic properties of this compound [87]. 

The anti-inflammatory properties of BCP have been extensively shown in different mouse models of disease. Bento and co-workers have demonstrated the beneficial effect of BCP treatment in an inflammatory bowel disease mouse model, in which BCP oral treatment mitigated TNF and Interleukin-1β (IL-1β) expression, reduced colon damage, and ameliorated disease score. To a mechanistic level, they showed these effects were at some degree dependent on peroxisome proliferator-activated receptor gamma (PPAR-γ) and CB2R activation [93]. In a very interesting study, Gertsch and co-workers reported that BCP selectively binds to CB2R acting as a full agonist, highlighting its potential therapeutic effects for inflammatory and painful states [94]. In an experimental autoimmune encephalomyelitis (EAE) mouse model, Alberti and co-workers have reported anti-inflammatory actions (i.e., reduced microglial activation and inducible nitric oxide synthase (iNOS) expression) of *Pterodon emarginatus* essential oil that is mainly enriched with BCP. Anti-inflammatory actions, in this case, contributed to attenuate neurological score and disease progression, being dependent on the control of T helper 1 (Th1) and Treg activity [95]. Later, the same authors demonstrated the effect of BCP in the experimental model of multiple sclerosis [96]. In fact, BCP extracted from *Cordia verbenacea* essential oil induced a markedly anti-inflammatory effect in panoply models in rats involving the attenuation of the abovementioned inflammatory molecules iNOS, TNF, and IL-2, as well as prostaglandin E2 (PGE2), and COX-2 [97]. Likewise, through anti-inflammatory pathways, BCP demonstrated a neuroprotective effect in a rat model of PD [98]. These are few very important examples of the beneficial and useful properties of caryophyllene. We agree with Sut and co-workers’ point-of-view that some of the considered old molecules, as sesquiterpenes, could possibly play an important role in drug discovery towards new discoveries [99]. 

### 3.2. D-Limonene 

Limonene, (4R)-1-methyl-4-prop-1-en-2-ylcyclohexene, is the most common monoterpene found in nature; for instance, in *Cannabis sativa* oilseed hemp named *Finola* and also in citrus oils, from orange, lemon, and tangerine [84]. Despite being found in *Cannabis sativa*, limonene does not interact with CB1R or CB2R [100]. Interestingly, D-limonene absorption and metabolism in animals is accelerated, and consequently it has a high rate of distribution and excretion. D-limonene metabolites have been detected in adipose tissue and mammary glands in a high concentration, although it has low toxicity [101]. This compound shows different pharmacological properties, which include anti-inflammatory, gastro-protective, anti-nociceptive, anti-tumor, and neuroprotective [102,103,104]. A recent study has demonstrated D-limonene anti-tumor activity (i.e., tumor cells decreased in proliferation and growth) in an animal model of chronic myeloid leukemia [102]. Moreover, D-limonene also showed anti-inflammatory activity by inhibiting pro-inflammatory mediators, leukocyte migration, and vascular permeability [105]. Regarding its activity on the gastrointestinal tract, there are different articles described in the literature. For instance, the same group described a gastric protection effect in rats with colon inflammation [103], and in an animal model of an ulcer induced by ethanol and indomethacin [106]. In addition, D-limonene-induced mucus production and IL-6, IL-1β, and TNF inhibition has been previously described [107]. Corroborating this data, Wang and colleagues demonstrated that limonene affected the intestinal microbiota of mice and enhanced the relative abundance of Lactobacillus, suggesting limonene direct effects on intestinal bacteria [108]. 

Limonene also inhibited nociceptive behavior induced by intraperitoneal acetic acid injection and plantar formalin [109]. In a complementary way, combined administration of limonene and β-ciclodextrin inhibited hyperalgesia in a chronic musculoskeletal pain model by downregulation c-FOS expression in the spinal cord [84]. Reinforcing this information, treatment with *Schinus terebinthifolius* essential oil—which is highly-concentrated in limonene—showed anti-hyperalgesic and anti-depressive effects in a neuropathic pain animal model [110]. At a different point-of-view, Smeriglio and colleagues reported the antioxidant and free radical scavenging properties of *Citrus lumia* oil, which is highly-concentrated in monoterpenes (e.g., 48.9% D-limonene and 18.2% linalool), suggesting an important preventive role in the genesis of oxidative stress-related pathologies [111]. In this context, a study conducted by Shin et al. showed that limonene decreased cell death, ROS levels, extracellular signal-regulated kinase phosphorylation, and overall inflammation in the brains and eyes of drosophila during Aβ42-induced neurotoxicity, a model of Alzheimer’s disease (AD) [104]. These and other authors have been studying limonene effects in the context of its impacts in the CNS. For instance, limonene has shown to exhibit anxiolytic effect increasing hippocampal dopamine levels and serotonin in the prefrontal cortex [75]. Considering the information above exposed, this is just one of the many compounds to be still addressed in this review that are natural and abundant in different plants, which could be used as potential therapeutics for diseases dependent on the inflammatory and oxidative-stress processes. 

### 3.3. Linalool 

Similar to limonene, linalool, 3,7-dimethylocta-1,6-dien-3-ol, is a monoterpene compound present in several medicinal plants and fruits, including *Cannabis sativa*, which has been widely used in the cosmetics and flavoring ingredients [112]. Linalool showed anti-inflammatory, anti-cancer, and anxiolytic effects [113,114,115]. The use of aromatherapy for the treatment of anxiety is disseminated among folk medicine. Accordingly, a study showed that linalool induced anxiolytic effects in mice by modulating GABAergic synaptic transmission [115]. Similarly to others terpenes, linalool showed anti-inflammatory activity, it prevented eosinophil migration, Th2-cytokines profile, and IgE concentration, in an asthma animal model. In addition, linalool inhibited iNOS expression, NF-κB (Nuclear factor kappa B) activation, inflammatory cells infiltration, and mucus hyper production during asthma progression [113]. Inflammation as well as oxidative stress are processes closely related to the progression of different CNS diseases, such as AD. In this context, a recent study demonstrated that linalool decreased ROS and lipid peroxidation levels, as well as improved mitochondrial morphology, membrane potential, and respiration, directly reducing the cell death rate due to oxidative stress [114]. Additionally, linalool showed neuroprotective effects on Aβ1–40-induced cognitive impairment in mice, which it was suggested to be mediated by inhibition of apoptosis and oxidative stress induced by Aβ-dependent Nrf2/HO-1 pathway activation [116].

Regarding to its potential anti-tumor activity, linalool induced apoptosis of cancer cells in vitro following the cancer-specific induction of oxidative stress, which was measured based on spontaneous hydroxyl radical production and delayed lipid peroxidation. Besides, mice in the high-dose linalool group exhibited a 55% reduction in average xenograft tumor weight compared to the control group [117]. Linalool has also reported to be protective against ultraviolet B (UVB)-induced tumor through inhibition of inflammation and angiogenesis signaling, as well as induction of apoptosis in the mouse skin [118]. Finally, a study showed that linalool reduced paclitaxel-induced acute pain in mice, which was antagonized by the direct injection of naloxone hydrochloride, suggesting opioid signaling modulation [119]. What can be appreciated so far, and will continue to be addressed, is the general ability of different terpenes to modulate inflammation and oxidative stress through different pathways, which in turn could be very useful to shed light to novel treatments for pain, cancer, autoimmune diseases, and CNS diseases that rely greatly on the impact of these processes. 

### 3.4. Terpineol 

Terpineol (2-(4-methylcyclohex-3-en-1-yl)propan-2-ol) is a volatile monoterpene alcohol present in the essential oil of *Cannabis sativa* [120], but also in several medicinal plants, such as *Punica granatum L.*, *Rosmarinus officinalis L*., and *Psidium guajava L.* Until this moment, there is no evidence in the literature about the interaction of terpineol with CBR. Nonetheless, this compound shows different pharmacological properties that include antinociceptive [121], antifungal [122], anti-inflammatory [123], and antidiarrheal [124]. Likewise, terpineol analgesic activity has been investigated in different animal models of pain. In this context, Oliveira and colleagues evaluated the effect of terpineol combined to β-cyclodextrin (βCD) (family of cyclic oligosaccharides with a wide variety of practical applications, including pharmacy, medicine, and foods) in an animal model of fibromyalgia. According to the authors, α-terpineol-βCD complex reduced nociceptive behavior induced by a chronic muscle pain model [121]. Still, this effect was mediated by activation of descending inhibitory pain system, since analgesic effect was reversed by systemic administration of naloxone (opioid antagonist), or ondansetron (5-HT3 antagonist) [121]. Additionally, terpineol has also been demonstrated to be a safe and effective drug for control of sarcoma-induced cancer pain in mice [125]. In a complementary way, terpineol could be investigated as preventive treatment for the development of dependence and of tolerance to opioid analgesics, since it attenuated the analgesic effect of morphine [126]. Thus, it is possible to suggest that terpineol alone, or combined to other drugs, could be an interesting target for development of new analgesics to control chronic pain symptoms. Besides, it could work as adjunctive therapy to morphine in order to reduce side effects related to treatment with opioid drugs. 

Terpineol showed not only antinociceptive but also neuroprotective properties, since improved memory impairment in rats exposed to transient bilateral common carotid artery occlusion. The underlying mechanisms described comprise the facilitation of LTP and suppression of lipid peroxidation, in the hippocampus [127]. In accordance, *Abies koreana* essential oil (terpenoids-rich oil, including terpineol) enhanced memory of mice submitted to scopolamine-induced amnesia [128]. Regarding it anti-inflammatory properties, terpineol has also been investigated for the treatment of allergic inflammation and asthma because decreased leucocyte migration and TNF levels. Furthermore, terpinen-4-ol and α-terpineol were found to suppress the production of inflammatory mediators (e.g., NF-κB, p38, ERK, and MAPK signaling pathways) in lipopolysaccharide (LPS)-stimulated human macrophages [129]. Altogether, data supports that terpineol should be better investigated in order to characterize its neuroprotective effects found in cerebral ischemia-related memory impairment and possibly be extended to other neurological conditions, such as seizures, migraine, Parkinson’s disease, as well as to clarify its anti-inflammatory potential. 

Terpineol properties go beyond, it has previously been shown antifungal properties against *Penicillium digitatum* because it disrupts fungi cell wall allowing the leakage of intracellular components [130]. In agreement with this, tea tree oil’s antibacterial and antifungal properties were attributed mainly to 1,8-cineol, methyl eugenol, and terpinen-4-ol [131]. Recently, Chaudhari and co-authors reported the efficacy of α-terpineol loaded chitosan nanoemulsion (α-TCsNe) to control AFB1, a secondary metabolite produced by *Aspergillus flavus* and *Aspergillus parasiticus* fungi [122]. Included in miscellaneous actions, in addition to bactericidal and antifungal activities, terpineol has been recognized as algaecide [132] and by its natural repellent activity against *Tribolium castaneum* (H.) [133]. Finally, this monoterpenoid exhibited strong anti-proliferative activity on cancer cell lines [134], as well as it inhibited growth of tumor cells trough modulation of NF-κB signaling pathway [135]. Thus, it is possible hypothesize that terpineol as a versatile compound with a wide variety of beneficial effects could be a possible venue for the development of new antibiotics, antifungal, and anticancer agents. 

### 3.5. Terpinene

Gamma-terpinene, 1-methyl-4-propan-2-ylcyclohexa-1,4-diene, is a monoterpene structurally similar to 1.8-cineol, being both found in the essential oils of *Cannabis sativa* and several other plants including the *Eucalyptus genus* (Myrtaceae), *Cupressus cashmeriana*, *Lippia microphylla*, *Lavandula angustifolia*, and *Citrus myrtifolia* [136,137,138,139,140,141]. Gamma-terpinene is very well described in the literature as an anti-inflammatory, antimicrobial, analgesic, and anticancer agent [136,137,142,143,144]. A recent study demonstrated that γ-terpinene reduced some inflammatory parameters, such as edema and inflammatory cell infiltration during tests in experimental models of inflammation, namely phlogistic agent-induced paw edema, acetic acid-induced microvascular permeability, carrageenan-induced peritonitis, and lipopolysaccharide-induced acute lung injury [145]. In addition, another study assessed the effect of γ-terpinene on pro- and anti-inflammatory macrophage production of cytokines in an animal model. The authors reported that γ-terpinene significantly increased the production of IL-10, which was dependent on PGE2 production since effects were reversed by COX-2 inhibitor nimesulide [146].

Besides the anti-inflammatory action, Assmann and colleagues described the anti-tumor activity and some of the possible underlying mechanisms of the *Melaleuca alternifolia* essential oil, which is composed of three major compounds terpinen-4-ol (41.98%), γ-terpinene (20.15%), and α-terpinene (9.85%), on MCF-7 breast cancer cells [147,148]. Authors reported γ-terpinene potential cytotoxic activity by decreasing breast cancer cells viability. Effects were observed in the early stages of apoptosis, such as increased BAX/BCL-2 genes ratio and increased cell arresting to S phase of the cycle [148]. Antimicrobial activity has been tested as well; *Melaleuca* spp. plants demonstrated effects against a wide range of gram-positive and gram-negative bacteria, fungi, and yeasts. Impressively, *Melaleuca thymifolia* volatile oil exhibits higher antimicrobial activity than *gentamicin* and *streptomycin* against *Staphylococcus aureus* [131]. Considering the exposed, it is feasible to suggest that γ-terpinene could server as natural immunomodulatory agent with antioxidant, antimicrobial, and anticancer properties that could be useful therapeutically.

### 3.6. Alpha (α)- and β-Pinene 

Alpha-pinene is considered a natural compound present not only in *Cannabis sativa* but also in essential oils of many aromatic plants, such as *Lavender angustifolia*, *Rosmarinus officinalis*, and coniferous trees [149]. Alpha-pinene is a bicyclo[3.1.1]hept-2-ene that contains a reactive 4-membered ring structure and exhibits antioxidant, antimicrobial, anti-tumor, hypnotic, and anxiolytic activities [83,120,150,151,152]. There are different biological properties described to α-pinene, as well as essential oils containing this compound have been used to treat several diseases [153], although no affinity towards CBRs have been described [154]. Alpha-pinene has been extensively investigated in the last years for its medicinal properties that include sedative, hypnotic, and anxiolytic [152,155]. In this context, Yang and colleagues demonstrated that α-pinene interacts with GABA_A_/benzodiazepine receptors prolonging its synaptic transmission, significantly increasing the duration of non-rapid eye movement sleep (NREMS), and reducing sleep latency [151]. The beneficial effects of α-pinene are also extended to convulsions [80,81], ischemic stroke [82], and schizophrenia [156]. Besides, α-pinene also showed neuroprotective effects that might be related to its antioxidant properties, which include being able to decrease malondialdehyde and hydrogen peroxide levels while increasing catalase and peroxidase activity. A study has reported that rats exposed to pentylenetetrazol (PTZ)-induced convulsions submitted to α-pinene intraperitoneal (i.p.) administration presented both initiation time delayed and reduced duration of myoclonic and tonic-clonic seizures, following PTZ injection [81]. Another study suggested that α-pinene appears to be devoid of anticonvulsant action, since only β-pinene affected the intensity of seizures and time of death of PTZ-treated mice [80]. Further, it was suggested that α-pinene might serve as potential therapeutics for schizophrenia since it possibly suppresses neuronal activity. However, it has also been demonstrated that inhalation of α-pinene inhibits dizocilpine (MK-801)-induced schizophrenia-like behavioral abnormalities in mice [156]. Lastly, α-pinene mitigated learning and memory loss induced by scopolamine in mice. The underlying mechanisms reported were increased choline acetyltransferase messenger RNA (mRNA) expression in the cortex and increased antioxidant enzyme levels (e.g., HO-1 and manganese superoxide dismutase (MnSOD)) in the hippocampus through activation of Nrf2 [157].

Beyond neuroprotection, the cytoprotective and antinociceptive properties of α-pinene have been previously described. Regarding the former, studies were conducted using peptic ulcer, ultraviolet A radiation (UVA) irradiation, and aspirin-induced cytotoxicity models [158,159,160]. In details, α-pinene was able to prevent UVA-induced loss of mitochondrial membrane potential, lipid peroxidation, DNA damages, and ROS generation [158]. Likewise, α-pinene inhibited UVA-induced activation of pro-angiogenesis factors (e.g., iNOS and vascular endothelial growth factor (VEGF)), as well as blocked expression of inflammatory mediators (e.g., TNF, IL-6, and COX-2) and apoptotic mediators (e.g., Bax, Bcl-2, caspase-3, and caspase- 9) in mouse skin submitted to UVA-irradiation at the rate of 10 J/cm2/day, for 10 days [159]. In contrast, α-pinene promoted cytoxicity, and consequently cancer cells apoptosis by increasing activity of caspase-3 in human ovarian cancer cells (PA-1) [161]. In this sense, another study showed that α-pinene was also able to inhibit human hepatoma tumor progression by inducing G2/M phase cell cycle arrest [162]. Regarding α-pinene antinociceptive effects, it was previously demonstrated its beneficial potential in capsaicin-induced dental pulp nociception [163], xylene-induced ear edema, and formalin-inflamed hind paw models [164]. In this context, α-pinene exhibited significantly anti-inflammatory and analgesic effects through inhibition of COX-2. Moreover, the analgesic effect of α-pinene on capsaicin-induced pulp nociception was blocked by co-administration with bicuculline or naloxone, thus suggesting that this effect could be mediated, at least in part, by interaction with GABA-A and μ-opioid receptors [163].

Related to α-pinene, another important monoterpene present in different *Cannabis sativa L*. varieties is β-pinene, which can also be found in many plants essential oils and obtained commercially by distillation or by α-pinene conversion [165,166]. Literature describes β-pinene antimicrobial and antioxidant activity [167], as well as its derivatives have been associated to anticancer, anticoagulation, and antimalarial effects. Additionally, β-pinene showed repellent activity against *Tribolium castaneum,* which is a beetle species from the *Tenebrionidae* family that is also a powerful invertebrate system for molecular genetics studies. Looking for the mechanism by which β-pinene mediated this repellent activity; authors reported that exposition to this compound alters the gene expression, namely Grd (which encodes GABA receptor), Ace1 (which encodes class A acetylcholinesterase) and Hiscl2 (which encodes histamine-gated chloride channel subunit 2) [168]. However, according to Pajaro-Castro and colleagues, β-pinene showed little ability to dock on proteins associated with neurotransmission process in the *Tribolium castaneum* [168]. Even though the β-pinene-induced repellent effect still remains to be fully addressed, it seems feasible to be considered that β-pinene monoterpene could act on different insect and mammalian receptors associated with neurotransmission. For instance, Guzmán-Gutiérrez and co-authors attributed to *Litsea glaucescens* essential oil (being β-pinene and linalool the two main active principles) antidepressant-like and sedative-like properties [169]. Posteriorly, the same group evaluated the mechanisms related to antidepressant effect of the essential oil compounds. In brief and focused on β-pinene, adult male ICR mice were pre-treated with (1*S*)-(−)-β-pinene (100 mg/kg) and exposed to forced swimming test (FST). Results showed that β-pinene, as well as imipramine (control drug), decreased the immobility time of mice when compared with control in the FST. Furthermore, administration of 5-HT_1A_ receptor antagonist prevented the antidepressant-like of β-pinene, demonstrating that this compound could interact with the serotonergic system. Likewise, β-pinene anti-immobility effects were also prevented by propranolol (β-receptor antagonist), neurotoxin DSP-4 (noradrenergic neurotoxin), and SCH23390 (a D1 receptor antagonist), suggesting its possible interactions with the adrenergic and dopaminergic system as well [170].

The use of β-pinene as an antitumor, as well as antiviral and antifungal agent has also been explored. Regarding the former, β-pinene-based thiazole derivatives were investigated as antineoplastic agents in vitro. Twenty-four β-pinene-based thiazole derivatives were synthesized and 5 g compound showed cytotoxic against three different cancer cell lines (Hela, CT-26, and SMMC-7721). Cytotoxic effect have been described to be mediated by action in the following signaling pathways: i) increased ROS activity, ii) loss of mitochondrial membrane potential, and iii) altered expression of Bax/Bcl-2, ultimately provoking cell injury and even cell death [171]. Concerning its antiviral and antifungal activity, it was shown its beneficial effects against *Rhizopus stolonifer* (the common bread mold) and *Absidia coerulea* fungi, as well as against herpes simplex virus type 1 (HSV-1), in vitro [172,173]. In fact, β-pinene reduced HSV-1 viral infectivity through interaction with free virus particles by 100% in a dose-dependent manner [174]. Similarly, β-pinene was able to reduce *Candida* biofilm adhesion through molecular interaction mainly with delta-14-sterol reductase–enzyme, which is related to metabolic pathway leading to cholesterol biosynthesis; thus, an effective target for antifungal drugs development [175,176]. Interestingly, when combined with commercial antimicrobial ciprofloxacin, both β-pinene and α-pinene demonstrated synergistic activity against methicillin-resistant *Staphylococcus aureus* [177]. Summarizing, here we describe, the antioxidant, anti-inflammatory, and immunomodulatory activity of both pinenes. Importantly, the neuromodulatory role that α-pinene and β-pinene are able to play could be used to shed light on innovative approaches to treat a variety of neurological conditions.

### 3.7. β-Elemene

β-elemene (1-methyl-1-vinyl-2,4-diisopropenyl-cyclohexane) is a derivative terpenoid found in *Cannabis sativa*, which may arise due to oxidation or due to thermal- or UV-induced rearrangements during processing or storage [85,178,179]. However, β-elemene is present not only in *Cannabis sativa* but also from *Curcuma rhizome,* and it is commonly used in traditional Chinese medicine due to its anticancer properties with no reported severe side effects [180]. In this way, this compound has been extensively studied as an anticancer agent in vitro and in vivo and has been demonstrated to be a promising drug for the treatment of a wide variety of tumors [181,182,183,184,185,186]. Among the challenges associated to cancer treatment, it is the development of multidrug resistance (MDR), which negatively impacts the effect of chemotherapy drugs, and consequently treatment success. It was previously proposed that one of the viable solutions to overcome MDR is to combine two chemotherapeutic drugs, acting synergistically to target multiple key pathways to inhibit tumor progression [187,188]. In this context, the combination of β-elemene with other chemotherapeutic agents (i.e., cisplatin and doxorubicin) and other therapeutic adjuvant has demonstrated great potential to inhibit tumor cells and tumor growth. According to Li and colleagues, β-elemene and cisplatin combined chemotherapy treatment is one of the most important approaches available for lung cancer therapy in China. Besides, the China Food and Drug Administration has approved it for the treatment of different tumors, such as brain, ovary, prostate, breast, lung, liver, and colon [189,190,191]. Additionally, when associated to hyperthermia β-elemene significantly inhibited growth of adenocarcinoma human alveolar basal epithelial cells A549 cells in a dose-dependent manner, when compared to β-elemene treatment alone [182]. Mechanistically, the exposition of A549 cells to hyperthermia plus β-elemene significantly increased mRNA expression of cyclin-dependent kinase inhibitor p21 that ultimately induced cell apoptosis [182]. Another approach to try overcoming unsuccessful chemotherapy is the nanotechnology-based drug delivery system, which could improve pharmacokinetics of chemotherapeutic agents [192]. These carriers encompass a broad range of dispersion systems (i.e., polymeric micelles, liposomes, and dendrimers) that protect against drug degradation, promote sustained release, and reduce side effects [192]. Thus, different studies evaluated the therapeutic effects of β-elemene co-loaded with chemotherapy drugs: i) cisplatin in co-loaded liposomes [193]; ii) doxorubicin (DOX) in pH-sensitive nanostructured lipid carriers (DOX/β-elemene Hyd NLCs) [194]; iii) cabazitaxel in complex liposome [195]. In summary, these reports described that β-elemene co-loaded with lower doses of chemotherapy drugs was able to induce toxicity effects against tumor while retaining a similar therapeutic effect of the drug by itself, demonstrating synergistic effect of the compounds. Corroborating, β-elemene was also described as a radiosensitizer producing DNA damage and inhibition of DNA repair, as well as increased apoptosis. Beta-elemene was also able to inhibit the activation of the Prx1-NFκB-HIF-1α axis, a key regulator whereby tumor cells adapt to radiation therapy and hypoxia [196]. Beta-elemene was also shown to inhibited monocyte chemoattractant protein-1 (MCP-1) secretion, a macrophage recruitment chemokine that contributes to cancer cells metastasis [197]. Altogether, these reports demonstrate the possible mechanisms behind β-elemene anticancer activity and suggest different ways to incorporate this compound into current clinical therapies.

Besides the very promising anticancer activity, it has been reported in the literature a variety of other beneficial effects attributed to β-elemene. Li and co-authors, for instance, provided evidence of β-elemene beneficial effects for atherosclerosis treatment [198]. In this study, apoE homozygous deficient mice were fed a high-fat diet during four weeks followed by β-elemene (135 mg/kg) oral gavage administration for another 12 weeks. Beta-elemene treatment significantly reduced lipid areas of atherosclerotic plaques and aortic root lesion sizes and necrotic core, basically by boosting antioxidant enzymes while decreasing inflammatory cytokines levels. [198]. In a different study, β-elemene exerted retino-protective effect by downregulation of hypoxia-inducible factor–1alpha (HIF-1α), VEGF, iNOS, and pro-inflammatory mediators during diabetes progression in a streptozotocin (STZ)-induced rat model [199]. Finally, the potential application of β-elemene in an EAE animal model was tested, in which mice were treated from day one after induction with β-elemene (20 mg/kg, i.p.) until the end of experiment. Beta-elemene reduced IFN-γ and IL-17 levels and completely blocked EAE onset and the severity of clinical symptoms. Furthermore, β-elemene inhibited IL-17, IFN-γ, ROR-γT, and T-bet mRNA expression in the optic nerve of EAE mice [200]. If we start to appreciate the bigger picture, it is possible to note that as the other terpenes here described so far, β-elemene shows the ability to modulate essential biological functions, such as inflammation, oxidative stress, immunology response, cell division, as well as endothelial regulation. Beneficial properties of this compound have been studied to a mechanistically level highlighting it as a promising tool for the treatment of relevant diseases, but there are many venues that still remain to be explored.

### 3.8. β-Ocimene and Camphene

Beta-ocimene (3,7-dimethyl-1,3,6-octatriene) is acyclic monoterpene that serves as a chemical cue to attract natural enemies of phytophagous insect in several plant species, including *Cannabis sativa* [85]. Booth et al. demonstrated using the variety ‘*Finola*’ of *Cannabis sativa* oilseeds that the most abundant monoterpenes found were myrcene, (+)-α-pinene, (−)-limonene, (+)-β-pinene, terpinolene, and (*E*)-β-ocimene [85]. Farré-Armengol and colleagues demonstrated that the emissions of β-ocimene in flowers follow marked temporal and spatial patterns of emission, which are typical from floral volatile organic compound (VOC) emissions that are involved in pollinator attraction [201]. Another study reported that a monoecious cultivar (*Futura 75*) and a dioecious one (*Finola*) of *Cannabis sativa* tested in a mountain area in Alps, Italy (elevation: 1100 meters above sea level, during the growing season 2018) showed particular phytochemical behavior. For instance, inflorescences from *Finola* variety were characterized by higher concentrations of β-ocimene and α-terpinolene, while α- and β-pinene accompanied by extremely high β-myrcene were found as predominant in *Futura* variety indicating that geographical provenience should be considered for a specific medicinal use of *Cannabis sativa* [202]. Currently, at least three beneficial properties have been described in the literature for this compound, such as antitumor, antifungal, and anticonvulsant [203,204], but mechanisms underlying the biological activity of this compound remain poorly explored.

Camphene (2,2-dimethyl-3-methylidenebicyclo(2.2.1)heptane) is a cyclic monoterpene present in *Cannabis* inflorescence in low titer but abundant in the essential oil of *Thymus vulgaris* that showed some pharmacological activities, such as expectorant, spasmolytic, and antimicrobial [205]. Camphene showed fumigant and contact toxicity against *Liposcelis bostrychophila* and *Tribolium castaneum* insects. Furthermore, it presented moderate repellent effect to *T. castaneum* while showed attractant effect to *Liposcelis bostrychophila*, [206]. Extending these observations, Benelli et al. showed that camphene inhibited *Helicoverpa armigera* and *Spodoptera litura*—key polyphagous insects pest—with a lethal dose (LC50) of 10.64 and 6.28 μg/mL, respectively, confirming the promising potential as a botanical insecticide [207,208]. Altogether, these findings strongly support the use of camphene as an eco-friendly and effective insecticidal agent. More recently, Souza and co-authors evaluated the anti-*Mycobacterium tuberculosis* activity of 17 novel synthesized thiosemicarbazones derived from (−)-camphene, *in vitro*. Overall, the majority of the tested compounds exhibited significant inhibitory effects on the *Mycobacterium tuberculosis* growth, with minimal inhibitory concentrations (MIC) values ranged from 3.9 to > 250 μg/mL [209]. Although there are not as much reports about β-ocimene and camphene as was described to the other compounds here reviewed thus far, their repellent and/or insecticide activity seem to be promising.

### 3.9. Nerolidol

Nerolidol ((6E)-3,7,11-trimethyldodeca-1,6,10-trien-3-ol), also known as peruviol, is a noncyclic sesquiterpene alkene alcohol common to citrus peels, *Piper claussenianum*, *Baccharis dracunculifolia*, and *Cannabis* plant [210]. Previously, it was demonstrated its inhibitory effect on the growth of *Leishmania braziliensis* promastigotes. Importantly, ultra-structural observation of nerolidol-treated parasites by STM showed mitochondria morphological alterations in the, nuclear chromatin and flagellar pocket along with cell shrinkage. In this same study, authors demonstrated some nerolidol mechanisms of action that included loss of mitochondrial membrane potential, phosphatidylserine exposure, and DNA degradation [211]. These evidences have been further exploited and extended in a study showing that nerolidol also inhibited *Leishmania amazonensis* amastigotes and promastigotes (with IC50 values between 2.6 and 3.0 M), indicating substantial accumulation of nerolidol in the cell membrane [212]. What is also relevant to this topic are the findings demonstrating the antiparasitic activity of nerolidol in mice infected with adult stages of *Schistosoma mansoni*. Authors showed that nerolidol (100, 200, or 400 mg/kg oral route) inhibited worm burden and egg production, directly associated with tegumental damage, although nerolidol showed low efficacy in mice harboring juvenile schistosomes. [213]. Substantiating, Baldissera et al. reported that nerolidol-loaded nanospheres mitigated the *Trypanosoma evansi*-induced cytotoxic and genotoxic effects in the rodent brain tissue during infection by upregulating NO levels; thus, preventing DNA damage and cell death [210]. Such results strongly support that nerolidol (a food additive and safe molecule) is an effective antiparasitic agent and could potentially display anti-inflammatory properties.

Regarding its potential anti-inflammatory and/or immunomodulatory activity, there are a number of studies using different cell-based and rodent models, which here we summarize. A study has shown that nerolidol blocked LPS-induced acute kidney injury by inhibiting the TLR4/NF-κB signaling pathway. Specifically, nerolidol markedly prevented the rise of nitrogen and creatinine levels in LPS-treated rats, and also inhibited the increase of inflammatory mediators, like TNF, IL-1β, and NF-κB in LPS-treated NRK-52E cells [214]. Further, de Souza et al. demonstrated that nerolidol nanoencapsulation improved its anti-inflammatory effect on zymosan-induced arthritis in mice. Importantly, under the conditions assessed the formulation did not demonstrated cytotoxicity in J774 cell line [215]. A study has also shown the immunomodulatory actions of trans-nerolidol on the efficacy of doxorubicin in breast cancer cells and in a breast tumor mouse model. The compound increased doxorubicin accumulation into MDA-MB-231 and MCF7 breast cancer cells while blocked cell migration ability, in vitro [216]. In addition, nerolidol demonstrated positive effects on cyclophosphamide (CYP)-induced neuroinflammation, oxidative stress, and cognitive impairment, as well as prevented structural abnormalities in the hippocampus and cortex regions of rodents [217]. The same authors also showed using in silico approach that nerolidol binds into Nrf2 pocket domain—a key nuclear factor that regulates the expression of antioxidant proteins [217], as previously addressed in this review. In summary, authors concluded that nerolidol could be a prospective therapeutic molecule that can mitigate CYP-induced neurotoxic signs through regulation of Nrf2 and NF-κB pathway [217], although further studies are needed to confirm this neuroprotective hypothesis. Lastly, cardioprotective effects have been suggested to this compound by the same research group. They previously evaluated nerolidol cardioprotective potential as an oral treatment against CYP-induced cardiotoxicity in mice. Nerolidol inhibited cardiac inflammation, oxidative stress, cardiac apoptosis, and cardiac fibrosis, as well as ultra-structural changes leading to cardiac dysfunction induced by cyclophosphamide [218]. Corroborating, Asaikumar et al. showed that nerolidol inhibited isoproterenol-induced myocardial damage in rats [219]. Here we reviewed the most described and better-explored activities of the nerolidol, which are antiparasitic, anti-inflammatory and/or immunomodulatory, and cardioprotective.

### 3.10. Euphol

Euphol is a tetracyclic triterpene usually extracted in alcoholic preparations due to its chemical structure and therefore affinity for this solvent. Even though it is not a major compound of the *Cannabis* plant, one could find a few chemically structure similarities in between the euphol molecule and a couple of cannabinoids derivate, such as CBD and CBN [220]. In fact, euphol is the major compound found in different plant species from the Euphorbiaceae family [221], including *Euphorbia resinifera, Euphorbia nerifolia, Euphorbia bivonae, Euphorbia umbellata,* and *Euphorbia tirucalli.* Regarding the latest cited *Euphorbia tirucalli*, it is a common plant found in Brazil and by far the most studied species from the Euphorbia family in concern to its major compound: euphol. Studies on euphol chemical structure using x-ray crystallographic, Fourier transform-ion cyclotron resonance mass spectrometry, tandem mass spectrometry, and gas chromatography coupled mass spectrometry, as well as its quantitative determination in the rat plasma by liquid chromatography-tandem mass spectrometry allowed a better understanding of this compound chemical and biological behavior [222,223,224]. Importantly, ethnopharmacology evidences have lead and contributed to studies on the anticancer and anti-inflammatory effects of this triterpene compound, as by many years the plants from this family have been used as folk phytomedicine to treat tumors and inflammation states [221]. Although, limited studies on antiviral, antiparasitic [225,226], antimicrobial, and antifungal activities of euphol have been recently reported. In our point, the most interesting aspect of a recent study is the finding that euphol can modulate the immune system by inducing cytokine production, namely IL-4, IL-3, and IL-2; thereby, influencing the Th1/Th2 balance [227]. These results could help to explain and support many of the previous described actions of euphol as an anti-inflammatory compound that will be discussed later. That being established, the two most described activities of this compound are the antitumor and the anti-inflammatory. The former is the primary and the most reported activity in the literature, being described for different *Euphorbia* species as well as cancer cell types while the latter is more recent; however, better studied in terms of mechanism of action. For instance, *Euphorbia tirucalli*-derived euphol beneficial effects against many cancer cell lines was previously tested and described. These cell lines included tumor cells from breast, head and neck, colon, glioma, prostate, epidermis, lung, bladder, melanoma, esophagus, ovary, and pancreas. Euphol cytotoxicity effect was observed against all cancer cell lines being very pronounced in this last cited, in which inhibited proliferation, motility, and colony formation as well [228]. Likewise, *Euphorbia umbellata*-derived euphol exhibited cytotoxic effects against K-562 leukemia cell line; being suggested that the main mechanism of action was apoptosis induction [229]. Other mechanisms of action proposed to euphol cytotoxic activity against breast and glioblastoma tumor cell lines included CDK2 downregulation whilst upregulates p21- and p27-CDK inhibitors and autophagy induction/facilitation, respectively [230,231]. Despite of its beneficial anticancer effect, very recently a study has suggested that euphol, along with sitosterol and lupeol, could cause hepatotoxicity by inducing significant increase in alanine aminotransferase, aspartate aminotransferase, and total bilirubin levels in rats treated sub-chronically with *Euphorbia bivonae* extract [232]. That consists of one report showing potential toxic actions of this compound in one species while there are many other enlightening reports describing its safety and its beneficial use to treat inflammatory diseases. Reports from a group in the south of Brazil coordinated by Professor Calixto in the early 2010s have described many of this compound uses towards inflammatory diseases management, as well as possible mechanisms of action. The earliest report described its anti-inflammatory actions on a mouse model of colitis, in which this compound inhibited important inflammatory cytokine production in the colon tissue (e.g., IL-1β, MCP-1, TNF, and IL-6); besides, the inhibition of adhesion molecules (i.e., selectins and integrins) [233]. A second study reported that euphol also inhibits inflammatory mediators and lymphocyte function-associated antigen-1 (LFA-1) integrin in the CNS, as it did in the periphery. At this time, euphol blocked Th17 myelin-specific cell migration with an overall benefic effect of reducing the severity and development of EAE, a multiple sclerosis model [234]. Later, it was described its beneficial action in a skin-inflammation mouse model induced by 12-O-tetradecanoylphorbol-13-acetate (TPA), corroborating early 2000s findings described by a Japanese group, and further extending the understanding about euphol mechanisms of action by showing that it inhibits TPA-induced protein kinase C (PKC) isoforms [235,236]. Later, PKC inhibition was again implicated in mediating euphol anti-inflammatory effects, as well as CB1R and CB2R in mouse models of inflammatory (e.g., PGE2-, carrageenan-, and complete Freund’s Adjuvant (CFA)-induced) and neuropathic (e.g., spared nerve injury (SNI)-, paclitaxel-, and B16F10 melanoma cells-induced hypersensitivity) pain [237,238]. Notably, cannabinoid-mediated anti-inflammatory actions involve suppression of inflammatory cytokines, MAPKs pathway activation, and modulation of TNF and NF-κB [220], all pathways in which euphol has been demonstrated to effective. Euphol has the potential to be a very attractive anti-inflammatory molecule that works through the cannabinoid system but evidence shows that it definitely can go beyond that.

### 3.11. Citral

Citral, (2E)-3,7-dimethylocta-2,6-dienal, is the main compound of essential oils that have been used mainly in popular medicine in eastern countries. It is the major compound extracted from *Cymbopogon citratus*, popularly known as lemongrass, but it can also be extracted from different plants including lemon myrtle and *Lindera citriodora* [239]. This essential oil has been used as ingredient in foods because of its lemon-like fragrance. However, citral has gained attention in the last years due to its antimicrobial properties against *Cronobacter sakazakii,* a foodborne pathogen clinically associated to neonatal infections such as meningitis, septicemia, and/or necrotizing enteritis [240,241]. Its reported antimicrobial activity also extends to *Staphylococcus aureus* [242], *Candida albicans* [243], *Enterobacter cloacae* [244], *Listeria monocytogenes* [245], *Aeromonas spp*. [246], and *Streptococcus pyogenes* [247]. In this context, Yang and colleagues recently demonstrated that when combined with cinnamaldehyde, citral changed cecal microbiota composition of non-vaccinated and vaccinated broiler chickens, reducing the incidence and severity of necrotic enteritis induced by coccidiosis [239]. This is in accordance with another finding, in which citral was able to affect mouse intestinal microbiota, enhancing the relative abundance of *Lactobacillus* [108]. From these evidences, it was possible suggest that citral could be an important molecule for development of new antibiotic and antifungal drugs, especially because until the moment there is no evidence of relevant toxicity and side effects related to its accumulation in tissues and delayed excretion [248]. However, Sharma and co-authors have well highlighted that strategies are required to increase citral stability, which could facilitate its applications [249].

Citral has also been recognized by its anti-inflammatory actions in animal models of acute lung injury [250], carrageenan-induced paw edema and croton oil-induced ear edema [251], segmental glomerulosclerosis [252], pleurisy [253], and peritonitis [254]. In this context, citral inhibited LPS-induced myeloperoxidase (MPO) activity, TNF, COX-2, and IL-8 expression, as well as NF-κB activation via PPAR-γ [254,255]. In accordance, Shen and colleagues demonstrated that GW9662 PPAR-γ antagonist reversed the anti-inflammatory response mediated by citral. Additionally, citral showed antioxidant properties linked to inhibition of Nrf2 pathway early activation, oxidative stress, and apoptosis [252]. More recently, Gonçalves and colleagues demonstrated that citral immunomodulatory property appears to be related to its ability to modulate CB2R, TLR4 and TLR2/dectin-1, as well as signaling pathways downstream of CBR and TLRs activation, including ATP-dependent K+ channels [256]. The antioxidant activity of this compound was also shown when co-administrated with aspirin in rat small intestine epithelial cells, in which it regulated superoxide dismutase (SOD) and glutathione (GSH) enzymes, significantly decreasing the aspirin-induced cell death [257]. Importantly, a link between its antioxidant and antinociceptive activity has been shown in an animal model of rheumatoid arthritis. Citral has promoted a decrease in oxidative stress parameters and induced antinociceptive effects through serotonergic communication at spinal the spinal cord level [227]. In fact, the citral antinociceptive activity is among the broad variety of beneficial effects already contemplated in the literature. When combined to other analgesics as naproxen, citral increased their antinociceptive activity as well significantly inhibited naproxen-induced gastric injury [258]. However, citral showed high volatility, low solubility in water, and consequent low bioavailability, which could limit its use. One possible solution could be the combination of citral with β-cyclodextrin and hydroxypropyl-β-cyclodextrin, which in turn demonstrated antihyperalgesic and anti-inflammatory activity [253]. Here we could suggest that citral should be better investigated in order to identify its possible clinical application for the treatment of chronic pain conditions, such as peripheral neuropathy, fibromyalgia, complex regional pain syndrome (CRPS) and lumbar chronic pain.

Beyond, citral attracted scientists’ attention towards its anticancer properties in a variety of cancer types, such as melanoma [259], colon cancer [260], and breast cancer [261]. Bayala and co-authors provided evidence about *Cymbopogon citratus and Cymbopogon gigantescus* essential oil cytotoxic activity, which have citral as its major component and significantly decreased prostate and glioblastoma cancer cell survival [262]. In addition, citral showed cytotoxic effect in non-tumoral HaCaT and tumoral A431 cells, inhibiting NO production even at the lowest concentration tested [263]. Regarding the possible mechanisms underlying its antiproliferative effects, it has been reported MARK4 and a Ser/Thr kinase inhibition. Of note, aberrant expression or dysregulation of these proteins are linked with cancer development, such as hepatocellular carcinoma, glioma, and metastatic breast carcinomas [264,265]. Other mechanisms also comprise apoptosis induction and downregulation of the aldehyde dehydrogenase activity—a reactive protein overexpressed during cancer progression and therapy resistance [266,267]. From this, it was previously suggested that citral could work as aldehyde dehydrogenase inhibitor, and consequently as adjuvant therapy for treatment of some types of cancer [268]. In order to improve citral solubility and delivery without enhancing toxic effects in vivo, Nordin and colleagues incorporated citral into a nanostructured lipid carrier (NLC) and evaluated its in vitro anti-cancer effects. Initially, they showed that NLC as a drug delivery system for citral has the potential to sustain drug release without inducing any toxicity [269]. Then, they showed that NLC-citral regulated apoptosis, cell cycle, and metastasis signaling, all key signaling pathways related to cancer development [261]. In addition, citral was pointed as a potential effective additive to chemotherapeutic treatment [270,271]. Thus, when combined with hyperthermia intraperitoneal chemotherapy (HIPEC) and pirarubicin for colorectal cancer, citral increased the HIPEC efficacy by enhancing chemo-drug penetration and consequently its intracellular concentration. Furthermore, it was described a safe alternative that decreased the chemo-drug dose necessary to induce antiproliferative effect reducing possible side effects [271]. Still, this natural compound showed chemoprotective actions in hairless (HRS/J) mice exposed to UVB irradiation for 24 weeks, a model of skin carcinogenesis. Mechanisms involved in citral chemoprotective effect not surprisingly included oxidative stress and inflammatory cytokines inhibition and increased skin cell apoptosis [272]. It has been previously described that citral mediated antiproliferative effects through p53activation, ROS- and mitochondrial-mediated apoptosis, as well as by NO depletion and interference with cell proliferation-related signaling pathways [259,260]. Collectively, these set of data here gathered suggests that citral represents an important molecule for the management of different types of cancer and highlights the possibility of translational application as a novel treatment alone or in combination with other chemotherapeutic drugs.

### 3.12. Celastrol

Celastrol, 2R,4aS,6aR,6aS,14aS,14bR-10-hydroxy-2,4a,6a,6a,9,14a-hexamethyl-11-oxo-1,3,4,5,6,1 3,14, 14b-octahydropicene-2-carboxylic acid, is a pentacyclic triterpenoid isolated from *Tripterygium wilfordii* root extracts and used in traditional Chinese medicine for treatment of chronic diseases, including neurodegenerative disorders (e.g., amyotrophic lateral sclerosis, AD, and PD), type 2 diabetes, obesity, atherosclerosis, cancer, inflammatory and autoimmune diseases (e.g., systemic lupus erythematosus, multiple sclerosis, inflammatory bowel disease (IBD), psoriasis, and rheumatoid arthritis (RA) [273,274,275]. In fact, this natural compound has been cited in a wide variety of reports describing its antioxidant [276,277], and anti-inflammatory action [278,279] through inhibition of NF-κB signaling pathway [280]. In details, this last study demonstrated that celastrol significantly blocked COX-2 expression, IL-8 and ICAM-1, as well as IL-1β-induced PGE2 through inhibition of NF-κB in a Graves’ ophthalmopathy model using orbital fibroblasts [281]. Here are a few more examples of this extent literature about celastrol anti-inflammatory effects. Kim and co-authors demonstrated that celastrol inhibited LPS-stimulated NO generation, PGE2, iNOS, and COX-2, in RAW264·7 cells. In this same study, authors have reported that celastrol inhibited LPS-induced inflammatory cytokines production and also protected mice from TPA-induced ear edema by inhibiting MPO activity and the production of inflammatory mediators [278]. In addition, celastrol inhibited CFA-induced arthritis rat model via modulation of *i)* inflammatory cytokines (i.e., IL-17, IL-6, and IFN-γ) in response to the disease-related antigens, *ii)* IL-6/IL-17-related transcription factor STAT3, *iii)* cyclic citrullinated- and Bhsp65-peptides directed antibodies, and *iv)* MMP-9 and phospho-ERK activity, supporting the use of celastrol as an adjunct (along with conventional drugs) or alternative approach for the RA treatment [279]. Aside from the anti-inflammatory effect, also relevant are the findings demonstrating celastrol antitumor activity in a variety of human tumor cell types. Data previously suggested that celastrol represents a promising agent for the management of human tumor cell lines, such as triple negative breast cancer [282], leukemia [283,284], carcinoma [285] and lung cancer [286]. In terms of mechanisms, a study based on pharmacological and biochemical approaches has shown that celastrol inhibited cell proliferation and induce apoptosis through JNK activation, AKT suppression, and anti-apoptotic proteins downregulation [287].

Celastrol potential beneficial effects on the CNS have also been previously reported. Kiaei et al. described that celastrol improved weight loss, motor performance, and delayed the onset of motor neuron degeneration in the G93A SOD1 transgenic amyotrophic lateral sclerosis (ALS) mouse model. Celastrol increased HSP70 while mitigated iNOS, TNF, cluster of differentiation 40 (CD40), and GFAP proteins expression in the lumbar spinal cord of G93A mice [288]. Celastrol effects on HSPs have also been reported to play a key neuroprotective role in defense against misfolded proteins and aggregation-prone proteins [289]. Speaking of protein aggregation, celastrol was reported to inhibit amyloid beta aggregation, the main toxin to be accounted for AD initiation and progression [276,281]. Based on these facts, we could suggest that celastrol might represent a useful molecule to treat neurodegenerative diseases with an inflammatory background. In spite of that, celastrol use is still limited by its low water solubility, reduced oral bioavailability, and side effects reducing its therapeutic potential [290]. Different structure modifications or encapsulation solutions must be studied to overcome this problem.

### 3.13. Falcarinol

Falcarinol—(3R,9Z)-heptadeca-1,9-dien-4,6-diyn-3-ol)—also named panaxynol or carotatoxin is found in carrots, parsley, celery, and *Panax ginseng* [291]. This natural compound has been cited in a wide variety of reports describing its antineoplastic [292] and anti-inflammatory properties [293]. Besides, falcarinol has been also investigated as pharmacological tool for treatment of cardiovascular and metabolic diseases. Regarding the latter, it is know that serum high molecular weight (HMW) adiponectin values are inversely correlated with the presence of metabolic syndrome, and consequently linked to pathogenesis of insulin resistance, type 2 diabetes, and cardiovascular diseases [294]. In this sense, Takagi and colleagues demonstrated that falcarinol restored FoxO1 and increased C/EBPα levels (transcription factors that positively regulate adiponectin gene transcription), resulting in HMW adiponectin secretion by 3T3-L1 adipocytes treated with palmitic acid, an obesity model in vitro [295]. In addition, falcarinol also reduced endoplasmic reticulum (ER) stress, C/EBP homologous protein (CHOP) protein and ROS levels, as well as decreased inflammatory adipokine-induced MCP-1 [295]. Still in this scenario, the association of chronic inflammatory disorders and/or systemic diseases to microbiota dysbiosis has been gaining attention [296]. Importantly, a study previously showed that the beneficial effects of falcarinol and falcarindiol rely on its ability of changing the composition of low abundant gut-microbiota members. In this study, the ability of falcarinol to regulate microbiota was allied to its ability to reduce the incidence of neoplastic lesions [292]. In this cancer scenario, the mucosa-associated bacterial population as the fecal microbiota plays an important role in colon carcinogenesis, the second most commonly diagnosed cancer with high incidence, morbidity, and mortality [296,297]. That being established, Kobaek-Larson and co-authors have reported that daily diet supplementation with falcarinol and falcarindiol decreased the number of neoplastic lesions and polyps growth rate in the colon of azoxymethane-treated rats [298]. Recently, this same group demonstrated the chemopreventive effect of a special diet supplemented with falcarinol and falcarindiol on colorectal precancerous lesions in a dose-dependent manner; besides, this effect was mainly mediated by inhibition of NF-κB and its downstream inflammatory markers, especially COX-2 [299]. Anticarcinogenic properties of falcarinol were also demonstrated in cancer stem-like cells (CSCs), in which it played an essential role in tumor occurrence, evolution, metastasis, recurrence, and therapeutic resistance [300], as well as in non-small cell lung cancer (NSCLC) [301]. Essentially, falcarinol eliminated CSC population in NSCLC and abolished lung tumor formation in mice via HSP90 (a molecular chaperone of numerous oncoproteins) modulation [302]. Falcarinol anticancer activity also extends to leukemia [303], breast cancer [304], hepatocarcinoma [305], renal carcinoma [306], and glioma [307]. For instance, mechanisms pointed to explain its ability to induce cell cycle arrest, thus, its anticarcinogenic properties on human promyelocytic leukemia cell growth are PKCδ proteolytic cleavage, caspase-3 activation, and PARP degradation [303].

In a different context, falcarinol has been also reported to be a facilitator of type 1 hypersensitivity and atopic dermatitis [308]. On the other hand, Leonti and colleagues showed that falcarinol is not an allergen itself; however, it facilitates sensitization by other allergens, since it aggravated histamine-induced edema reactions in skin prick tests. In this study, similar effects were obtained with Rimonabant^®^ (a CB1R inverse antagonist), implying that falcarinol-induced dermatitis could be related to CB1R antagonism in keratinocytes [291]. Despite that falcarinol has been related to allergic reactions, it has also been shown to induce anti-inflammatory responses in a couple of different models. Falcarinol promoted a reduced cell infiltration in a LPS-induced reduction in intestinal barrier context [293]. In addition, falcarinol was able to induce Nrf2-mediated resolution of inflamed macrophage-induced cardiomyocyte hypertrophy [309]. Collectively, data here presented provided information about falcarinol crucial positive effects on pathological conditions, such as metabolic diseases, cardiovascular diseases, and cancer. However, we consider that for the development of possible therapeutic tools underlying mechanisms as well as toxicity, and bioavailability needs be better investigated.

### 3.14. Salvinorin A

The trans-neoclerodane diterpenoid salvinorin A is a short-acting highly-selective kappa opioid receptor agonist and consequently the primary psychoactive component of *Salvia divinorum* (psychoactive herb used in magic-ritual contexts by Mazateca Indians in Mexico) [310]. In agreement, eight healthy hallucinogen-using adults exposed to inhalation of 16 doses of *Salvia divinorum* showed dose-related dissociative effects and impairments in recall/recognition memory tests [311]. Given the fact that salvinorin A highly interacts with opioid receptors, it has been considered an emerging target for next-generation of analgesics. In addition, salvinorin A showed hallucinogen effects similarly to lysergic acid diethylamide (LSD) [312,313]. Walentiny and colleagues demonstrated that salvinorin A administration induced pronounced hypolocomotion and antinociception (and to a lesser extent, hypothermia) effects in the tetrad assay, which were reverted by the administration of kappa opioid receptor (KOR) selective antagonist but not by CB1R antagonist Rimonabant^®^ [310]. Moreover, rats exposed to sciatic nerve ligature neuropathic pain model and treated with salvinorin A directly in the insular cortex showed antinociceptive behavior. However, in contrast with Walentiny and colleagues, the analgesic effect of salvinorin A in this case was reverted by selective KOR and CB1R antagonists [314]. In accordance with this finding, daily treatment with salvinorin A significantly decreased formalin-induced mechanical allodynia at days three and seven in a KOR and CB1R dependent manner, without inducing CB1R-related adverse effects. Electrophysiological experiments in vivo also showed that repeated salvinorin A treatment completely normalized neuronal activity following formalin injection, as well as it reduced formalin-evoked glial and microglial activation at the spinal cord level [315]. Nonetheless, unlike other opioid ligands, salvinorin A showed short duration of action and centrally mediated side-effects limiting its usefulness [316,317,318], justifying the development of new salvinorin A analogues [319]. In this context, novel analogue β-tetrahydropyran salvinorin B attenuated acute nociceptive and inflammatory pain, as well as mechanical and cold allodynia in the PTX-induced neuropathic pain model [319]. On the other hand, mesyl salvinorin B (a KOR agonist) showed moderated antinociceptive effect when compared to salvinorin A in warm-water (50 °C) tail withdrawal and intraplantar formaldehyde (2%) tests. However, it mitigated cocaine-induced hyperactivity and behavioral sensitization, without affecting aversion, sedation, anxiety, or learning and memory impairment in rats [320]. Additionally, mesyl salvinorin B alone or associated with naltrexone prevented alcohol-induced deprivation effect in mice [321], which could represent an alternative tool for treatment of alcoholism in humans. Other salvinorin A analogues, such as p38, could also be effective for the treatment of gastrointestinal inflammation, since it demonstrated anti-inflammatory and analgesic effects in an experimental model of colitis [322]. Thus, these findings support the use of novel salvinorin A-like compounds and its analogues as possible pharmacological alternatives for pain relief, control of cocaine-seeking behavior, and alcoholism, as it seems to have potent CNS and anti-inflammatory actions.

Regarding these actions, the anti-inflammatory effects associated with salvinorin A also extend to cerebral hypoxia/ischemia [323,324,325,326]. Salvinorin A attenuated brain edema and inhibited neuronal death in hippocampal CA1 region, cortex, and striatum during forebrain ischemia model [325]. According to Dong and colleagues, rats submitted to middle cerebral artery occlusion and treated with salvinorin A one hour after reperfusion showed improvement of neurological severity score when compared to control groups. Additionally, salvinorin A reduced infarct volume and effectively protected cerebral vessels after ischemia/reperfusion. Importantly, human brain microvascular endothelial cells exposed to the oxygen glucose deprivation model and treated with salvinorin A were protected against ROS damage and decreased mitochondrial function (i.e., mitochondrial morphological changes and loss of membrane potential). The latter, highly regulated by AMPK and phosphorylation mitofusin-2 expression, both upregulated in response to salvinorin A treatment [327]. Salvinorin A also mitigated cerebral vasospasm through endothelial nitric oxide synthase (eNOS) and NO upregulation and ET-1 downregulation. At the same time, salvinorin A inhibited AQP4 protein expression—a member of a family of channel proteins that facilitate water transport and contribute to brain edema and neuro-disorders development [326,328]. Concerning still its actions in the CNS, salvinorin A effects on the mood were also investigated and linked to anxiolytic and antidepressant properties mediated by KOR, as well as the ECS [329]. In lieu of antidepressant properties, another study associated salvinorin A to depressive-like effects through dopamine signaling inhibition in the *nucleus accumbens* of rats [330]. Extending, dysphoria as well as depressant-like effects of salvinorin A were attributed to KOR-linked ERK activation, which in turn promoted dopamine transporter (DAT) phosphorylation, modulating dopamine neurotransmission [331]. Recently, Keasling and colleagues evaluated the effects of salvinolin, a new semisynthetic analog of salvinorin A, with mu opioid receptor affinity. In summary, salvinolin demonstrated good oral bioavailability and showed antidepressant-like effect that was blocked by the selective 5HT_1A_ antagonist WAY100635 [332]. Another derivative of salvinorin A, the 22-azido salvinorin A, also promoted an antidepressant-like effect linked to its ability of inhibiting monoamine oxidase (MAO) enzyme, as well as to its affinity for α1A, α1B, α1D adrenergic receptors beyond KOR [333]. Here, we could sense the staggering effects of salvinorin A and its analogues to modulate a variety of neurotransmission systems in the CNS.

The pharmacological effects of salvinorin A are not limited to CNS but also related to the respiratory system. Salvinorin A inhibited mast cell degranulation in the lung and consequently blocked airway hyperactivity induced by ovalbumin sensitization. Thus, the authors suggested that salvinorin A could represent a promising tool for the treatment of type 1 hypersensitivity and immune-mediated diseases [334]. Moreover, salvinorin A inhibited leukotriene production in inflammatory exudates, as well as it showed antipruritic effects mediated by KOR on compound 48/80-induced scratching behaviors in mice [335]. Findings here summarized provide evidence about the anti-inflammatory action of salvinorin A, and highlight this natural compound as a possible new tool for the treatment of inflammatory diseases.

### 3.15. Pristimerin

Pristimerin (20α-3-hydroxy-2-oxo-24-nor-friedela-1-10,3,5,7-tetraene-carboxylic acid-29-methyl ester) is a natural quinonoid triterpene isolated from the shrub families *Celastraceae* and *Hippocrateaceae*. It is a natural compound with cannabimimetic effects without direct interacting with CBR. For instance, pristimerin inhibited MAGL with high potency through a reversible mechanism [336]. It has been extensively investigated mainly by its inhibitory activity against cancer cell growth. Pristimerin inhibited Wnt/β-catenin signaling via GSK3β activation and Wnt gene suppression in colorectal cancer cells [337]. In addition, Yousef and colleagues demonstrated pristimerin anticancer activity on colon tumor cells associated to NF-κB signaling inhibition during the carcinogenic process [338,339]. Corroborating, this triterpenoid has also been shown to attenuated colitis-associated colon cancer by modulating NF-κB positive cells, as well as AKT/FOXO3a signaling pathway [340]. The transcription factor FOXO3 represents important target for cellular homeostasis, since it was able to regulate apoptosis, proliferation, cell cycle progression, and consequently tumorigenesis [341,342]. Pristimerin was also previously demonstrated to downregulate the PI3K/AKT/mTOR pathway playing a critical cytotoxic and anti-metastatic role in the progression of HCT-116 colorectal cancer cells in vitro and in vivo [343]. Finally, another study from Yousef and co-authors suggest that pristimerin downregulates phospho-EGF and -EGRF2 and its downstream signaling pathways, which represent a key mechanism involved in the proliferation of cancer malignant phenotypes [344,345].

The antiproliferative activity of pristimerin goes beyond colon-related cancers, it is extended to breast [346,347,348,349,350], melanomas [351], osteosarcoma [352], pancreatic [353,354], and prostate cancers [355,356,357,358]. Herein, we describe a few examples focusing on articles that have demonstrated potential mechanisms of action. Pristimerin anticancer activity against breast cancer cells was associated to ROS production and ASK1/JNK signaling pathway activation [346], as well as AKT signaling suppression [349,359]. Additionally, when combined to paclitaxel, pristimerin induced cell autophagy through inhibition of ERK1/2/p90RSK signaling—involved in cancer cell proliferation, differentiation, and migration [347,360]. Pristimerin-induced glioma overgrowth was dependent on AGO2 upregulation (a critical protein for tumorigenesis) and PTPN1 downregulation (a metabolism regulator oncogene reported to be aberrantly expressed in cancer cells) [361,362,363]. Furthermore, pristimerin induced glioma cell necrosis by promoting mitochondrial dysfunction, c-Jun activation, and consequently ROS overproduction [364]. It also inhibited the epidermal growth factor receptor (EGFR) protein expression during glioma cancer development [365,366]. Antiproliferative effects of pristimerin were investigated in oral squamous cell carcinoma cell lines as well. In this way, pristimerin showed more potent antiproliferative activity than chemotherapy drugs cisplatin and 5-fluorouracil. This effect was associated with inhibition of MAPK1/2 and PKB signaling pathways [367]. Pristimerin-induced apoptosis activity was also demonstrated in ovarian cancer cells via inhibition of AKT/NF-κB/mTOR signaling pathway [368]. Besides, few articles have reported pristimerin beneficial effects on prostate cancer. Its progression was reported to be prevented by pristimerin-induced inhibition on HIF-1α and SPHK-1, which stimulates different cellular processes including cell proliferation, cell survival, and angiogenesis [355,369]. Pristimerin also induced apoptosis of prostate cancer cells through activation of mitochondrial apoptotic pathway [358], ubiquitin-proteasomal degradation [357], and inhibition of proteasomal chymotrypsin-like activity (a complex associated with cell proliferation, apoptosis, and cancer progression) [370,371]. These summarized findings provide evidences regarding pristimerin antiproliferative and cytotoxic activity as well as clinical benefits for treatment of different types of cancer.

Finally, yet importantly, Tong and co-authors showed that pristimerin inhibited arthritic and cartilage inflammation, as well as bone damage in the joints of rats submitted to adjuvant arthritis. Pristimerin inhibited inflammatory cytokines and pSTAT3 and ROR-γt transcription factors, as well as Th17/Treg ratio favoring immune suppression [372]. In addition, anti-inflammatory properties of pristimerin included inhibition of inflammatory cytokine levels (e.g., IL-6, IL-17, IL-18, and IL-23), increase IL-10 expression, and mitigate NF-κB and MAPK signaling, showed during rheumatoid arthritis model and murine macrophages exposed to LPS [373]. In this sense, pristimerin seems able to interact with essential targets of the inflammatory and/or immune-mediated processes; and for this reason, it should further investigated regarding its potential ability to serve as a treatment of disorders related to the imbalance in the immune system, including autoimmune diseases.

## 4. Conclusions

**Figure 3:**
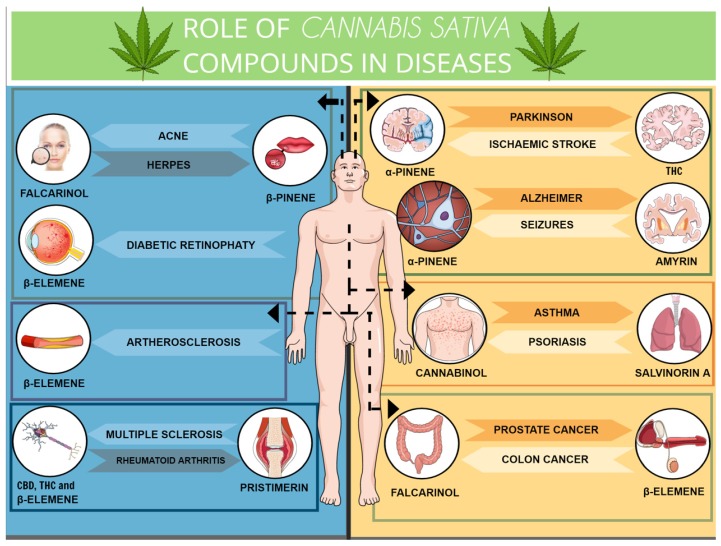
Role of *Cannabis sativa* compounds in diseases. The *Cannabis sativa* compounds have been proved useful for treatment of different diseases in the periphery and the CNS, as illustrated above. The CBD and THC actions in the CNS include immunomodulatory, neuroprotective, anxiolytic, and anticonvulsant, in addition to its potential effects on PD and multiple sclerosis control. Anticancer effects can be attributed to almost all *Cannabis sativa* compounds. This figure further illustrates the effect of terpenoids, cannabimimetic ligands, beyond the *Cannabis* plant in different pathological conditions, such as *Herpes* infection, diabetic retinopathy, psoriasis, asthma, AD, seizures, ischemic stroke, and others. Figure created using the Mind the Graph platform.

The reports here highlighted showed the complex and varied pharmacology of *Cannabis sativa*, particularly phytocannabinoids—typical terpenophenolic compounds—as well as plenty of non-cannabinoids second metabolites, such as monoterpene, sesquiterpene, and stilbenoids. Interestingly enough, there are an increasing number of studies on cannabimimetic ligands beyond the *Cannabis* plant, which can act as CBR agonists or antagonist, or ECS enzyme inhibitors. They are mainly terpenes including β-caryophyllene, D-limonene, terpineol, β-elemene, euphol, pristimerin, citral, and many others (Figure 3), which can play a key role in the modulation of different pathological conditions.

Herein, we describe that many of them share common properties, namely anti-inflammatory, analgesic, immunomodulatory, antiproliferative, and neuromodulatory. More specifically, the majority of these compounds seem to be acting on the same targets even though if in different pathological contexts (Table 3). We highlight the NF-κB, Nfr2, PPAR, COX-2, and CDKs proteins, just to name a few. Although there are many published preclinical studies demonstrating the beneficial effects of terpenes, there is an urge for detailed pharmacokinetic and pharmacodynamics characterization of these compounds. As the cannabinoids and the *Cannabis* plant appear to be the most recent great hope for the treatment of uncured diseases, the particular phytocannabinoid–terpenoid interaction—the so-called entourage effect—must be continuously investigated. Besides, clinical studies are sorely needed to confirm its efficacy and safety in humans; thus, we could finally have novel potential treatments for a number of diseases that for the time being remain poorly managed.

## Figures and Tables

**Table 1:** *Cannabis sativa* L. constituents by chemical class.

Chemical Class	Compounds
Δ9-THC types	23
Δ8-THC types	5
CBG types	16
CBC types	9
CBD types	7
CBND types	2
CBE types	5
CBL types	3
CBN types	11
CBT types	9
Miscellaneous types	30
Total cannabinoids	120
Total non-cannabinoids	445
Grand Total	565

THC, tetrahydrocannabinol; CBG, cannabigerol; CBC, cannabichromene; CBD, cannabidiol; CBND, cannabinodiol; CBE, cannabielsoin; CBL, cannabicyclol; CBN, cannabinol; CBT, cannabitriol, as previously described [20].

**Table 2:** CBD pharmacological actions on pathological conditions.

Research Themes	Main Findings	References
Alzheimer’s disease (AD)	CBD prevented expression of proteins involved with *tau* phosphorylation and AD progression. CBD showed therapeutic potential for AD-associated cognitive impairment.	[22,23]
Anti-inflammatory properties	CBD induced apoptosis and inhibited lipopolysaccharide-activated NF-κB and interferon-β/STAT inflammatory pathways in microglial cells; CBD protected oligodendrocytes progenitor cells from inflammatory-induced apoptosis.	[24]
Anxiety	CBD modulated anxiety responses partially through 5-HT_1A_-mediated neurotransmission, and demonstrated anxiolytic effects during a stimulated public speaking test; CBD action on limbic and paralimbic regions contributed to reduced autonomic arousal and subjective anxiety; CBD blocked anxiety-induced REM sleep alteration through anxiolytic properties.	[25,26]
Diabetes	CBD showed beneficial effects on glycemic control and cardiovascular dysfunction during diabetes.	[27]
Immunomodulatory effects	CBD modulated T-cell function and apoptotic signaling pathway.	[28]
Inflammatory bowel disease (IBD)	CBD attenuated intestinal inflammation and normalized motility in patients with IBD.	[29]
Cognitive impairments	CBD interacted with components of emotional memory processing and memory-rescuing, as well as attenuated THC-induced memory impairment effects.	[30]
Neuropathic pain	CBD inhibited chemotherapy-induced neuropathic pain.	[31,32]
Parkinson’s disease (PD)	CBD administration showed neuroprotective effects during PD progression.	[33]
Schizophrenia	CBD showed antipsychotic-like properties in schizophrenia, as well as prevented clinical social dysfunction, and inhibited psychomotor agitation.	[34,35]
Seizure/Epilepsy	CBD showed anticonvulsant effects in animal models of seizure and patients with refractory epilepsy. CBD was also described as safe and beneficial for the treatment of epileptic disorders.	[36,37,38,39]

CBD, cannabidiol; NF-κB, nuclear factor kappa B; STAT, signal transducer and activator of transcription protein family; 5-HT_1A_, serotonin 1A receptor; REM, rapid eye movement sleep; THC, tetrahydrocannabinol.

**Table 3:** The main findings about terpenoid compounds reviewed in the article.

Compound	Main Findings
β- and α-Caryophyllene	Antidepressant, anxiolytic, analgesic, anticonvulsant properties. Acetylcholinesterase (AChE) inhibitor.
D-Limonene	Anti-inflammatory, antinociceptive, gastroprotective, and neuroprotective effects.
Linalool	Anxiolytic, anticancer properties; neuroprotective effects against AD.
Terpineol	Analgesic activity in chronic pain conditions, such as fibromyalgia and cancer pain. Adjunctive therapy to morphine adopted in order to reduce its adverse effects. Preventive treatment for opioid analgesic dependence and tolerance.
Terpinene	Analgesic, antiproliferative, anti-inflammatory, and antimicrobial properties.
α-Pinene	Sedative, hypnotic, anti-seizure, anxiolytic, anticancer, and analgesic activities. Neuroprotective effects against memory loss.
β-Pinene	Antiviral, antifungal, anticancer, antimalarial, antidepressant properties.
β-Elemene	Anticancer and hypolipidemic compound. Potential treatment for demyelinating disease.
β-Ocimene	Antiproliferative, antifungal, and anticonvulsant properties.
Camphene	Eco-friendly botanical insecticide.
Nerolidol	Anti-inflammatory, anticancer, neuroprotective and antimicrobial effects.
Euphol	Antiviral, antiparasitic, antimicrobial, and antifungal activities.
Citral	Antimicrobial, anti-inflammatory, antinociceptive, and anticancer properties.
Celastrol	Anti-inflammatory and anticancer compound.
Falcarinol	Possible tool for treatment of cardiovascular diseases. Anticarcinogenic compound.
Salvinorin A	Psychoactive herb; anxiolytic, anti-inflammatory, and antidepressant effects. Alternative treatment for control of cocaine-seeking behavior and alcoholism. Promising tool for treatment of type 1 hypersensitivity.
Pristimerin	MGL inhibitor; anticancer and anti-metastatic effects.

AD, Alzheimer’s disease; MGL, monoacylglycerol lipase.

## Abbreviations

AC: Adenylyl Cyclase; AChE, Acetylcholinesterase; AFB1, Aflatoxin B1; Ago2, Argonaute 2; ALS, Amyotrophic lateral sclerosis; AMPK, AMP-activated protein kinase; apoE, Apolipoprotein E; Aβ, Amyloid-beta; BAX, BCL2 associated X protein; BCL2, B-cell lymphoma 2 protein; CD40, Cluster of differentiation 40; CHOP, C/EBP Homologous Protein; COX, Cyclooxygenase; COX-2, Cyclooxygenase-2; CNS, Central nervous system; CYP, Cyclophosphamide; DAT-1, Dopamine transporter; EAE, Experimental autoimmune encephalomyelitis; EGFR, Epidermal growth factor receptor; eNOS, Endothelial nitric oxide synthase; ET-1, Endothelin-1; FOXO3, Forkhead box O3; GABA, Gamma-aminobutyric acid; GPR18, G protein-coupled receptor 18; GPR55, G protein-coupled receptor 55; GPR119, G protein-coupled receptor 19; GSH, Glutathione; GSK-3β, Glycogen synthase kinase-3 beta; HIF-1α, Hypoxia-inducible factor – 1alpha; HMW, High molecular weight; HO1, Heme oxygenase-1; Hsp90, Heat shock protein 90; HSPs, Heat-shock proteins; i.p., Intraperitoneal; IBD, Inflammatory bowel disease; IFN-γ, Interferon-gamma; IgE, Immunoglobulin E; IL-1β, Interleukin -1β; IL-2, Interleukin – 2; IL-3, Interleukin – 3; IL-4, Interleukin – 4; IL-6, Interleukin – 6; IL-8, Interleukin – 8; IL-10, Interleukin – 10; IL-12, Interleukin – 12; IL-17, Interleukin – 17; IL-37, Interleukin – 37; iNOS, Inducible nitric oxide synthase; LFA-1, Lymphocyte function-associated antigen-1; LPS, Lipopolysaccharide; LOX, Lipoxygenase; LTP, Long term potentiation; MAO, Monoamine oxidase; MAPK, Mitogen-activated protein kinase; MARK4, Microtubule Affinity-Regulating Kinase 4; MCP-1, Monocyte chemoattractant protein-1; MMP-9, Matrix metallopeptidase – 9; MnSOD, Manganese superoxide dismutase; MPO, Myeloperoxidase; mTOR, Mammalian target of rapamycin; NF-κB, Nuclear factor kappa B; NO, Nitric oxide; NREMS, Non-rapid eye movement sleep; p90RSK, 90 kDa ribosomal S6 kinase; PARP, Poly ADP-ribose polymerase; PGE2, Prostaglandin E2; PI3K, Phosphoinositide 3-kinase; PKB, Protein kinase B; PKC, Protein kinase C; PPAR, Peroxisome proliferator-activated receptors; PPAR-γ, Peroxisome proliferator-activated receptor gamma; pSTAT3, Phosphorylated STAT3; PTX, Paclitaxel; PTZ, Pentylenetetrazole; RA, Rheumatoid arthritis; ROR-γt, Retinoid-related orphan receptor-γt; ROS, Reactive oxygen species; SNI, Spared nerve injury; SOD1, Superoxide dismutase – 1; SPHK1, Sphingosinekinase 1; Th1, T helper 1; Th2, T helper 2; TLR4, Toll-Like Receptor 4; TPA, 12-O-tetradecanoylphorbol-13-acetate; TRPM8, Transient receptor potential melastatin 8; TRP, Transient receptor potential; TRPV1, Transient receptor potential vanilloid 1; TRPV4, Transient receptor potential vanilloid 4; TNF, Tumor necrosis factor; UVA, Ultraviolet A radiation; VEGF, Vascular endothelial growth factor.
